# Microbial colonization and resistome dynamics in food processing environments of a newly opened pork cutting industry during 1.5 years of activity

**DOI:** 10.1186/s40168-021-01131-9

**Published:** 2021-10-14

**Authors:** José F. Cobo-Díaz, Adrián Alvarez-Molina, Elena A. Alexa, Calum J. Walsh, Oscar Mencía-Ares, Paula Puente-Gómez, Eleni Likotrafiti, Paula Fernández-Gómez, Bernardo Prieto, Fiona Crispie, Lorena Ruiz, Montserrat González-Raurich, Mercedes López, Miguel Prieto, Paul Cotter, Avelino Alvarez-Ordóñez

**Affiliations:** 1 Department of Food Hygiene and Technology, Universidad de León, León, Spain; 2 Present address: Microbiology Department, National University of Ireland, Galway, Ireland; 3 Teagasc Food Research Centre, Fermoy, Co. Cork, Ireland; 4 APC Microbiome Ireland, University College Cork, Cork, Ireland; 5 Department of Animal Health, Universidad de León, León, Spain; 6 Department of Food Science & Technology, International Hellenic University, Thessaloniki, Greece; 7 Institute of Food Science and Technology, Universidad de León, León, Spain; 8 Dairy Research Institute, Spanish National Research Council, Instituto de Productos Lácteos de Asturias-CSIC, Villaviciosa, Spain; 9 MicroHealth Group, Instituto de Investigación Sanitaria del Principado de Asturias (ISPA), 33011 Oviedo, Asturias Spain

**Keywords:** Metagenomics, Food processing environments, Antimicrobial resistance, Microbial ecology

## Abstract

**Background:**

The microorganisms that inhabit food processing environments (FPE) can strongly influence the associated food quality and safety. In particular, the possibility that FPE may act as a reservoir of antibiotic-resistant microorganisms, and a hotspot for the transmission of antibiotic resistance genes (ARGs) is a concern in meat processing plants. Here, we monitor microbial succession and resistome dynamics relating to FPE through a detailed analysis of a newly opened pork cutting plant over 1.5 years of activity.

**Results:**

We identified a relatively restricted principal microbiota dominated by *Pseudomonas* during the first 2 months, while a higher taxonomic diversity, an increased representation of other taxa (e.g., *Acinetobacter*, *Psychrobacter)*, and a certain degree of microbiome specialization on different surfaces was recorded later on. An increase in total abundance, alpha diversity, and β-dispersion of ARGs, which were predominantly assigned to *Acinetobacter* and associated with resistance to certain antimicrobials frequently used on pig farms of the region, was detected over time. Moreover, a sharp increase in the occurrence of extended-spectrum β-lactamase-producing *Enterobacteriaceae* and vancomycin-resistant *Enterococcaceae* was observed when cutting activities started. ARGs associated with resistance to β-lactams, tetracyclines, aminoglycosides, and sulphonamides frequently co-occurred, and mobile genetic elements (i.e., plasmids, integrons) and lateral gene transfer events were mainly detected at the later sampling times in drains.

**Conclusions:**

The observations made suggest that pig carcasses were a source of resistant bacteria that then colonized FPE and that drains, together with some food-contact surfaces, such as equipment and table surfaces, represented a reservoir for the spread of ARGs in the meat processing facility.

Video Abstract

**Supplementary Information:**

The online version contains supplementary material available at 10.1186/s40168-021-01131-9.

## Background

Food processing environments (FPE) can be an important source of microorganisms that cross-contaminate raw materials and processed foods, with important implications for food quality and safety [1]. Microorganisms can continuously access FPE through the entry of new raw materials and utensils, the flow of workers or the use of cleaning water, and some of them persist in the environment through the presence of harbourage sites in processing plants, surfaces that are difficult to clean or disinfect, or organic residues from food processing which can create microenvironments that support microbial growth [2, 3]. Moreover, specific taxa or lineages/strains that possess enhanced ability to form biofilms, survive sanitation, and/or mount adaptive stress responses are particularly well equipped to persist [4, 5].

The possibility that FPE may act as a reservoir of antibiotic-resistant (AR) microorganisms and a hotspot for antibiotic resistance genes (ARGs) transmission is a concern in meat processing plants [6]. The overuse of antibiotics as therapeutic, metaphylactic, or prophylactic agents in intensive rearing of food production animals, linked to the cross-contamination of meat with gut AR bacteria during evisceration and other dressing activities at slaughterhouses, may result in the introduction of AR microbes in meat processing plants [7]. In addition, certain biocides used for sanitation can induce the selection of enhanced resistance to other unrelated compounds, such as some antibiotics [6]. However, these poorly understood phenomena have not been directly confirmed in real industrial settings, and no single study has followed the emergence and establishment of AR bacteria in FPE from the moment a facility begins operations.

To date, culture-dependent analyses, coupled with the typing of recovered isolates through molecular techniques, have been widely used for unearthing routes of microbial cross-contamination to food and identifying episodes of persistence in FPE [8]. These approaches generally focus on mapping the distribution of specific environmentally transmitted pathogenic bacteria of high concern (e.g., *Listeria monocytogenes*) [9]. Recent advances in high-throughput sequencing technologies allow to perform larger-scale untargeted analyses of the resident microbial communities in FPE, which facilitate the tracking of a wider range of microbial agents and their associated gene repertoires [10]. However, with the exception of a few studies limited to the characterization of FPE through 16S rRNA gene amplicon sequencing [11–16], detailed culture-independent whole metagenome sequencing analyses have not yet been undertaken to characterize temporal shifts in the structure and resistome of their microbial populations.

Over the last decade, some pioneering studies characterized the microbiome of built environments and demonstrated its impact on the human microbiome and health status [17]. These initial experiences mainly focused on household domestic and hospital settings [18–22] but few studies have been done on food processing facilities [23]. The microbiome colonization of new environments is commonly characterized by an initial fluctuating period with high diversity values until the establishment of a more stable microbiome, as has been reported on infants [24], fermented foods [25], or building materials under high humidity conditions [20]. Considering these previous findings, we hypothesize that the microbiome of a newly established food manufacturing facility goes through waves of succession before becoming relatively stable and that daily processing and sanitation activities impact on the burden and composition of antimicrobial resistance determinants, leading to the establishment of reservoirs or hotspots of antimicrobial-resistant microorganisms in FPE. To test this hypothesis, here we present the results of a longitudinal 18-month survey of the bacterial and resistome diversity encountered within the FPE of a newly constructed pork cutting plant. In total, 1374 swab samples were collected from multiple surfaces on ten sampling visits, 229 sample pools were analysed through shotgun metagenomic sequencing, and a collection of 360 isolates from the *Enterobacteriaceae*, *Pseudomonadaceae*, *Enterococcaceae*, and *Staphylococcaceae* families was characterized to monitor the occurrence of phenotypes and genotypes associated with resistance to antibiotics of critical importance.

## Results

### The taxonomic diversity in FPE increased over time

**Fig. 1:**
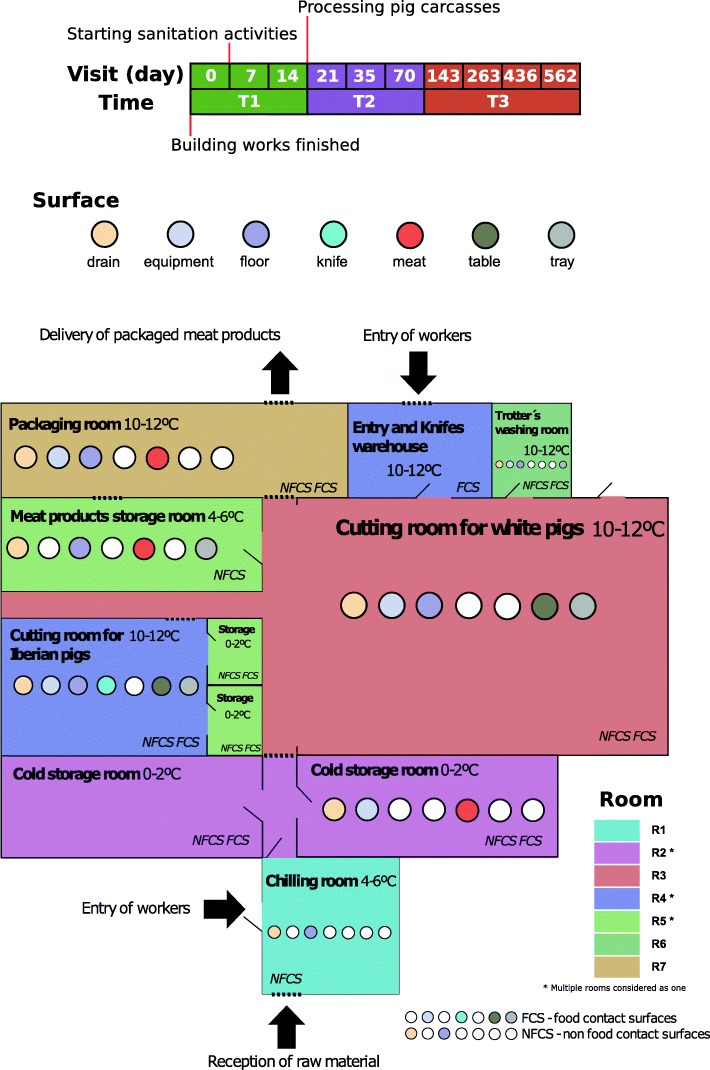
Schematic map of the meat processing facility and summarized information on the surfaces sampled per room. The 10 visits performed over 1.5 years were grouped in 3 time categories (indicated at the top of the figure). The different surfaces sampled at each processing room are indicated in the map, together with their classification as food contact surfaces (FCS) or non-food contact surfaces (NFCS). Asterisks in the “Room” legend indicate those room groups comprising more than one physical room

Samples were categorized to one of three temporal groups, i.e., T1 for samples before the processing plant became operational, T2 for samples within the first 2 months of operation (i.e., linked to short-term changes in the microbiome), and T3 for samples from 2 to 18 months of operation (i.e., associated with long-term changes in the microbiome) (Fig. 1).

**Fig. 2:**
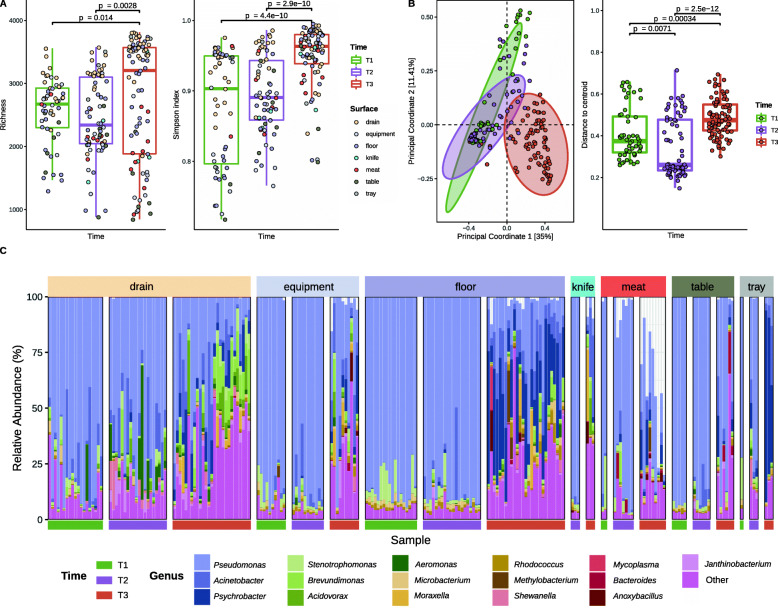
Changes in bacterial alpha diversity, beta diversity and bacterial taxonomy along the 1.5 years of activity. **A** Richness and Simpson indices and **B** Principal Coordinates Analysis, using Bray–Curtis distance, at species level for the 210 industry samples (*n* = 55 for T1, *n* = 70 for T2, *n* = 85 for T3). The centroid of each ellipse represents the group mean, and the shape is defined by the covariance within each group. Adonis test values are indicated in Table S1. Distance to the centroid values were employed to evaluate the homogeneity of variances within each group. Only significant *p* values (*p* < 0.05) obtained from the Wilcoxon signed-rank test analysis are indicated. **C** Barplot representing the relative abundance of the 16 most relevant bacterial genera (average total abundance > 1% or at least one sample with abundance > 15%). Other bacterial genera are grouped into “Other”. A sample is represented by each bar, and samples are grouped by Surface and Time groups, indicated at the top and bottom of the plot, respectively

Species richness and the Simpson index average values were similar on visits belonging to the same time category (see Additional file 1: Figure S1), but significantly increased (*p* < 0.05) from T1 and T2 to T3 (Fig. 2A). This pattern was particularly evident for drains, floors, equipment, and meat surfaces (see Additional file 1: Figure S2A), and for all processing rooms except R1 in the case of the Simpson index (see Additional file 1: Figure S2B). Drains were the surfaces with the highest alpha diversity regardless of the sampling period (see Additional file 1: Figure S3A), while no major differences were observed among rooms (see Additional file 1: Figure S3C).

Sampling time, surface type, and processing room had a significant influence on the taxonomic profile of samples (adonis, *p* = 0.002) and explained 22.8%, 14.5%, and 5.3% of the variation observed, respectively (see Additional file 2: Table S1). Ordination analyses resulted in T3 samples grouping separately from T1 samples, while T2 samples showed an intermediate profile between them (Fig. 2B). β-dispersion was significantly higher at T3 than at T1 and T2 (*p <* 0.001) (Fig. 2B). Drains were the surfaces with the highest β-dispersion, with drain samples from T1 and T2 clustering away from samples from other surfaces (see Additional file 1: Figure S3B). Differences in β-diversity were less evident between rooms, due to the stronger influence of sampling time and surface type (see Additional file 1: Figure S3D; Additional file 2: Table S1). Although T3 samples were collected over a broader time range, this is not the basis for the observed higher β-dispersion as the analysis by visit showed similar β-dispersion levels for all the visits corresponding to T3 (see Additional file 1: Figure S1).

Sixteen genera were identified as the most abundant colonizers of FPE, showing an average abundance of 1% or greater across all samples or an abundance of 15% or greater in at least one individual sample (Fig. 2C). Among them, *Pseudomonas*, *Acinetobacter*, and *Psychrobacter*, with average abundances of 56.7, 7.3, and 6.2%, respectively, were most dominant.

Among the most abundant taxa, a significant decrease (*p* < 0.001) was observed over time in the relative abundance of *Pseudomonas* and *Stenotrophomonas* (Fig. 2C; see Additional file 3), with this temporal decrease being significant (*p* < 0.05) across all surfaces for *Pseudomonas* (see Additional file 1: Figure S4A) and for equipment, floor, and meat samples for *Stenotrophomonas* (*p* < 0.05) (see Additional file 1: Figure S4B). In contrast, a significant increase (*p* < 0.001) in relative abundance over time was observed for *Acinetobacter*, *Psychrobacter*, *Brevundimonas*, and *Acidovorax*, among other genera (Fig. 2C; see Additional file 3), with the temporal increases being particularly marked for *Acinetobacter* on equipment, floors, and tables (see Additional file 1: Figure S4C); *Psychrobacter* on all surfaces except knives (see Additional file 1: Figure S4D); and *Brevundimonas* and *Acidovorax* on drains, equipment, and floors (see Additional file 1: Figure S4E and Figure S4F). Relative abundances and statistical information for all genera are shown in an additional file (see Additional file 3).

### The abundance and diversity of ARGs increased over time

**Fig. 3:**
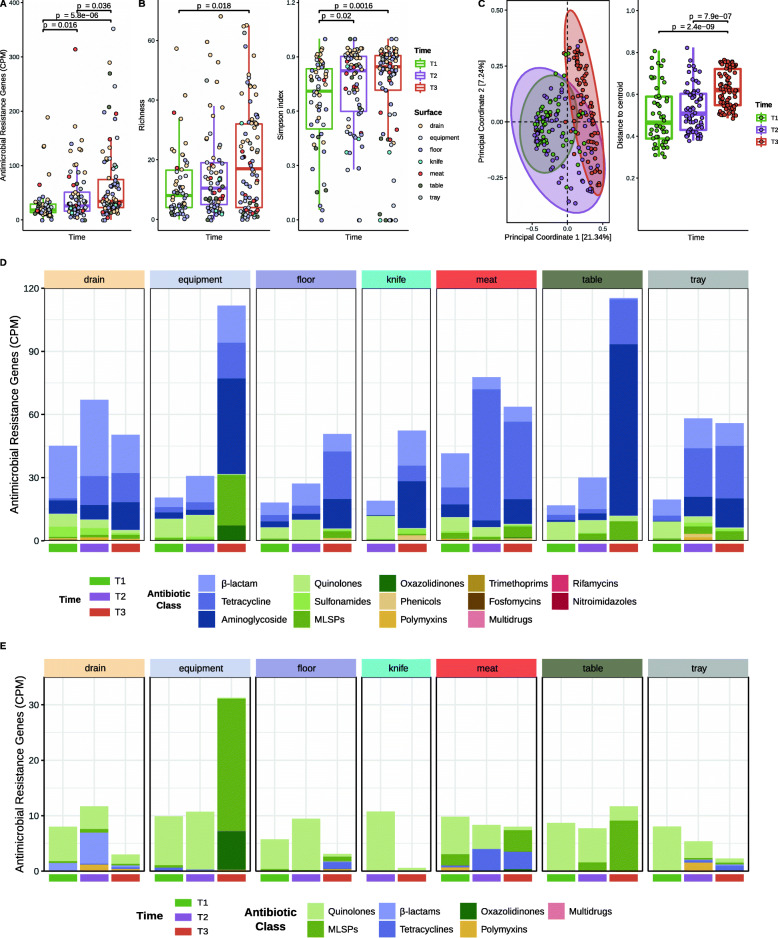
Resistome dynamic changes along time, as revealed through the evolution of alpha and beta diversity indices and ARG composition. **A** Antibiotic resistance genes (ARGs) counts per million reads (CPM); **B** richness and Simpson’s indices calculated with the ARG–CPM matrix; and **C** Principal Coordinates Analysis, using Bray–Curtis distance, at ARG level for the 210 industry samples (*n* = 55 for T1, *n* = 70 for T2, *n* = 85 for T3). The centroid of each ellipse represents the group mean, and the shape is defined by the covariance within each group. Adonis test values are indicated at Table S1. Distance to the centroid values were employed to evaluate the homogeneity of variances within each group. Only significant *p* values (*p* < 0.05) obtained from the Wilcoxon signed-rank test analysis are indicated. **D** Barplot of the 14 ARG classes detected and **E** the 7 ARG classes associated with resistance to antibiotics of critical importance calculated by adding ARG abundances according to the antibiotic classes they confer resistance to (Suppl. File 4). Each bar represents the average value for samples belonging to the same Surface and Time groups, indicated at the top and bottom of the plot, respectively. MLSP refers to macrolides, lincosamides, streptogramins, pleuromutilins

A significant increase (*p* < 0.05) in the total amount of ARGs was observed over time (Fig. 3A), mainly due to increased ARG counts per million reads (CPM) at T3 in equipment, floors, and tables and in R3, R5, and R6 (see Additional file 1: Figure S5A and S5D). Similarly, significant increases along time were observed for both richness and Simpson diversity indices (*p* < 0.05) (Fig. 3B). Drains had the highest resistome alpha diversity across all sampling times, while tables and equipment presented the lowest alpha diversity values at T3 (see Additional file 1: Figure S5B), and no major differences were found among processing rooms (see Additional file 1: Figure S5E).

In β-diversity analyses, T3 samples grouped separately from T1 and T2 samples, with T2 displaying an intermediate resistome profile (Fig. 3C), and showed a larger β-dispersion, with significantly higher average distance to the centroid than T1 and T2 samples (Fig. 3C). The effect of sampling time (adonis, *p <* 0.05) explained 11.7% of the variation, while surface type and processing room had a less marked effect on ordination, explaining up to 8.0% and 5.0% of the variation, respectively (see Additional file 1: Figure S5C and Figure S5F; see Additional file 2: Table S1).

### The resistome structure temporally evolved, and *Acinetobacter* and *Pseudomonas* were the most relevant carriers of ARGs

The most abundant ARGs were associated with resistance to β-lactams (27.2%), tetracyclines (26.9%), aminoglycosides (25.3%), and quinolones (7.5%), but the resistome composition evolved over time. Whereas the main group of ARGs detected at T1 was that related to resistance to β-lactams, followed by resistance to quinolones, a significant increase was observed from T1 and T2 to T3 in the relative abundance of ARGs related to resistance to aminoglycosides (*p* < 0.001), tetracyclines (*p* < 0.01) and antimicrobials of the MLSP group (macrolides, lincosamides, streptogramins, and pleuromutilins) (*p* < 0.05) (Fig. 3D; see Additional file 4). On the other hand, a significant decrease along time was found for ARGs linked to quinolone resistance (*p* < 0.001) (Fig. 3D; see Additional file 4). These global trends from T1 to T3 were generally observed on each surface and processing room (Fig. 3D; see Additional file 1: Figure S6A).

**Fig. 4:**
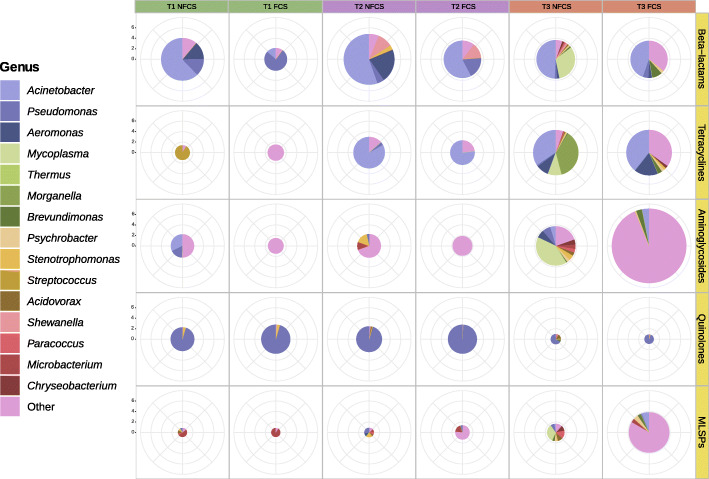
Taxonomic assignment of ARGs based on their alignment to contigs assembled from reads within the same sample. Pie charts representing the taxonomical distribution, at genus level, of ARGs for the 5 most abundant antibiotic classes. Colors indicate the average genera relative abundance (%) for samples belonging to the same Time group and to NFCS (non-food contact surfaces: drain and floor) or FCS (food contact surfaces: equipment, knife, table, and tray) groups. The size of each pie is proportional to the square root of the average value for the total ARGs detected in each group (Time–Surface type combination), indicated in sqrt(CPM) at y-axis. The genera on the legend are sorted by descending order according to the total ARGs detected

Up to 68.7% of the ARGs could be ascribed to contigs, which were taxonomically assigned to 72 different genera, among which *Acinetobacter* and *Pseudomonas* accounted for 28.1% and 9.3% of the total ARGs (Fig. 4; see Additional file 5). ARGs linked to β-lactam resistance were mainly assigned to *Acinetobacter* on NFCS, followed by *Aeromonas*, *Pseudomonas*, and *Shewanella*, and to *Acinetobacter* and *Pseudomonas* on FCS (Fig. 4; see Additional file 5). Quinolone-resistance–associated ARGs were mainly assigned to *Pseudomonas* on both FCS and NFCS (Fig. 4; see Additional file 5). Whereas a significant number of ARGs associated with resistance to aminoglycosides could not be classified at genus level, *Thermus*, *Mycoplasma*, *Pseudomonas*, and *Acinetobacter* were frequent carriers at T3 (Fig. 4; see Additional file 5). ARGs linked to resistance to tetracyclines were mainly assigned to *Acinetobacter* at T2 and to *Acinetobacter, Pseudomonas, Morganella*, and *Mycoplasma* at T3 (Fig. 4; see Additional file 5). ARGs of the MLSP group were mainly assigned to *Thermus* and *Pseudomonas* on FCS at T3, and to *Pseudomonas* and *Mycoplasma* at T3, on NFCS (Fig. 4; see Additional file 5).

Several different determinants of resistance to tetracyclines (*tet*(39), *tet*(H), *tet*(Q), *tet*(L)), β-lactams (*bla*_ROB-1_, *bla*_PAO_, *bla*_OXA-281_, *bla*_OXA-211_, *bla*_OXA-212_, *bla*_OXA-280_, *bla*_OXA-334_), and aminoglycosides (*aph(3)-Ib*, *aph(3)-Ia*, *aph(6)-Id, aadD, aadA6, ant(3)-Ia*) were among the most abundant ARGs (see Additional file 1: Figure S6B; see Additional file 6). The *oqxB* gene, associated with resistance to quinolones, was the most abundant determinant associated with resistance to critically important antibiotics (CIA), as defined by the World Health Organization [26]. Other less abundant ARGs associated with resistance to CIA mainly belonged to the quinolones ARG class, at T1 and T2, and the MLSP ARG class, on equipment, meat, and table surfaces at T3 (Fig. 3E). Notably, the total abundance of ARGs linked to resistance to CIA significantly decreased (*p* < 0.01) over time on drains and floors, and only increased on equipment surfaces (Fig. 3E; see Additional file 2: Table S2). Statistical information for all ARGs is shown in an additional file (see Additional file 6).

### The abundance of mobile genetic elements (MGE) was higher in drains and increased over time

**Fig. 5:**
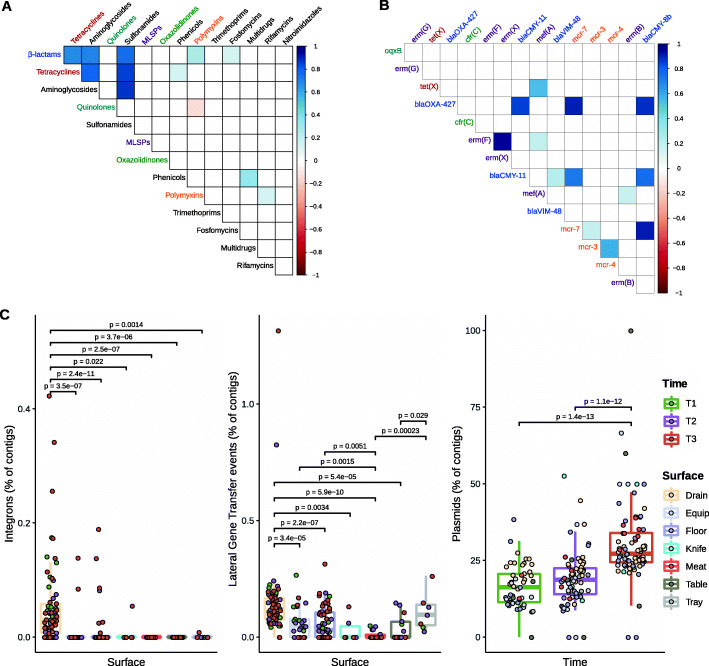
ARGs co-occurrence and mobilome distribution among surfaces and sampling times. **A** Correlogram of co-occurrence for the different ARG classes and ARGs detected in industry samples (*n* = 210). R-square values for the Pearson correlation between abundances of ARG classes (CPM) are indicated. Significant correlations (*p* < 0.05) are indicated in blue color (positive correlation) or red color (negative correlation). Gene names are colored according to the ARG classes they belong to, which are equally colored on Fig. 5A. **C** Mobilome characterization for FPE samples (*n* = 210), including the percentage of contigs carrying integrons and LGT events, or associated with plasmids. Contigs shorter than 1000 bp were removed before the mobilome analysis. Only significant *p* values (*p* < 0.05) obtained from the Wilcoxon signed-rank test analysis are indicated

Determinants from some ARG classes frequently coexisted together in FPE, as in the case for ARGs associated with resistance to β-lactams, tetracyclines, aminoglycosides, and sulphonamides (*R*^2^ > 0.6, *p* < 0.05) (Fig. 5A). Focusing on ARGs associated with resistance to CIA and with average abundances > 0.05 CPM, strong correlations were observed between *bla*_OXA-427_, *bla*_CMY-11_, *bla*_CMY-8b_ (linked to β-lactams resistance), and *mcr-7* (associated with polymyxin resistance) (*R*^2^ > 0.75, *p* < 0.05); and also between *erm(X)* and *erm(F)*, both associated with resistance to antibiotics of the MLSP group (*R*^2^ > 0.95, *p* < 0.05) (Fig. 5B). MGE and lateral gene transfer (LGT) events were significantly more abundant in drains than on other surfaces (*p* < 0.05), and the abundance of predicted plasmid-associated contigs was significantly higher at T3 than at T1 and T2 (*p* < 0.001) (Fig. 5C). Up to 57 integrons associated with ARGs were found, all associated with drain samples, with a clear dominance of T3 samples over T1 and T2 samples. All the identified ARG-carrying integrons contained *aadA* genes, associated with resistance to aminoglycosides (see Additional file 7).

### The occurrence of certain bacteria resistant to CIA sharply increased when pork cutting activities started

**Fig. 6:**
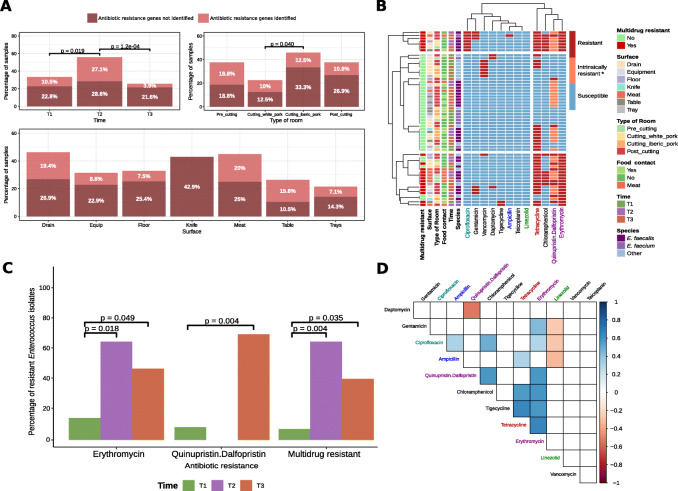
Characterization of AR phenotypes and genotypes for the isolates in the culture collection, with special emphasis on *Enterococcus* spp. strains. **A** Proportion of samples where antibiotic resistant isolates or ARGs were detected on FPE surfaces (*n* = 229). Color indicates detection of, at least, one phenotypically antibiotic-resistant isolate on selective agar plates (dark pink), or a positive PCR for one of the ARGs (light pink). Samples were grouped according to sampling time (*n* = 57 for T1, *n* = 70 for T2, and *n* = 102 for T3), processing room (*n* = 48 for Pre-cutting, *n* = 48 for Cutting_Iberian_pork, *n* = 40 for Cutting_white_pork and *n* = 93 for Post_cutting) and surface type (*n* = 67 for Drain, *n* = 35 for Equipment, *n* = 67 for Floor, *n* = 7 for Knife, *n* = 20 for Meat, *n* = 19 for Table, and *n* = 14 for Tray). Only significant differences (*p* < 0.05) obtained from the two-proportion Z test are represented. **B** Antibiotic resistance profile obtained for *Enterococcus* isolates from Sensititre panels. ECOFF values from EUCAST (see Additional file 2: Table S3) served as threshold to classify *Enterococcus* isolates (*n* = 58) as antibiotic resistant or susceptible. Isolates resistant to antibiotics of three different families were considered multidrug resistant. * *E. faecalis* is intrinsically resistant to quinupristin.dalfopristin. **C** Proportion of *Enterococcus* isolates (*n* = 14 for T1, *n* = 14 for T2, and *n* = 30 for T3) showing phenotypic resistance, with statistically significant changes along time as determined through the Fisher’s exact test. **D** Correlogram of phenotypic antibiotic resistance levels in *Enterococcus* isolates. Pearson correlation coefficients between the MIC values of those antibiotics included in the Sensititre panels (*n* = 12) were calculated. Only significant coefficients (*p* < 0.05) are represented in blue color (positive correlation) or red color (negative correlation). Antibiotics names are colored according to the ARG classes as on Fig. 5A. *E. fecalis* isolates (*n* = 2 for T1, *n* = 13 for T2, and *n* = 17 for T3) were removed from quinupristin.dalfopristin on (**C**) and (**D**) sections, due to their intrinsic resistance

Culture-dependent analyses undertaken on a collection of 360 isolates from FPE revealed that, at T1, only in 33.3% and 10.5% of the samples, the strains isolated showed resistance to at least one of the antibiotics evaluated in the phenotypic tests and carried at least one of the tested ARGs, respectively, while at T2, these values increased to 55.7% and 27.1% of the samples. However, these percentages decreased again to 25.5% and 3.9%, respectively, at T3 (Fig. 6A). These temporal patterns reflected the statistically significant increase from T1 to T2 of AR isolates from extended-spectrum β-lactamase (ESBL) producing *Enterobacteriaceae* (*p* < 0.05) and vancomycin-resistant *Enterococcaceae* (VRE) (*p* < 0.001) (see Additional file 1: Figure S7). Regarding surface type and processing room, drains and meat were the sample categories where the highest percentage of samples carrying AR microorganisms (46.3% and 45%, respectively) were found, although no significant differences across surfaces were observed, and the cutting room for Iberian pork showed a significantly higher proportion of samples containing AR bacteria than the cutting room for white pork (Fig. 6A).

The results obtained for a subset of 58 *Enterococcus* spp. isolates subjected to testing with a wider range of antibiotics showed that multidrug resistance profiles were mainly found among strains from meat and NFCS (Fig. 6B). A significantly higher (*p* < 0.05) representation of *Enterococcus* isolates showing erythromycin or multidrug resistance was recorded at T2 and T3 relative to T1 (Fig. 6C). Moreover, significant positive correlations were found between the minimum inhibitory concentrations observed for several pairs of antibiotics (e.g., erythromycin, tetracycline, and tigecycline; *R*^2^ > 0.55, *p* < 0.05) (Fig. 6D).

### A microbiome specialization took place on different surfaces at long term

**Fig. 7:**
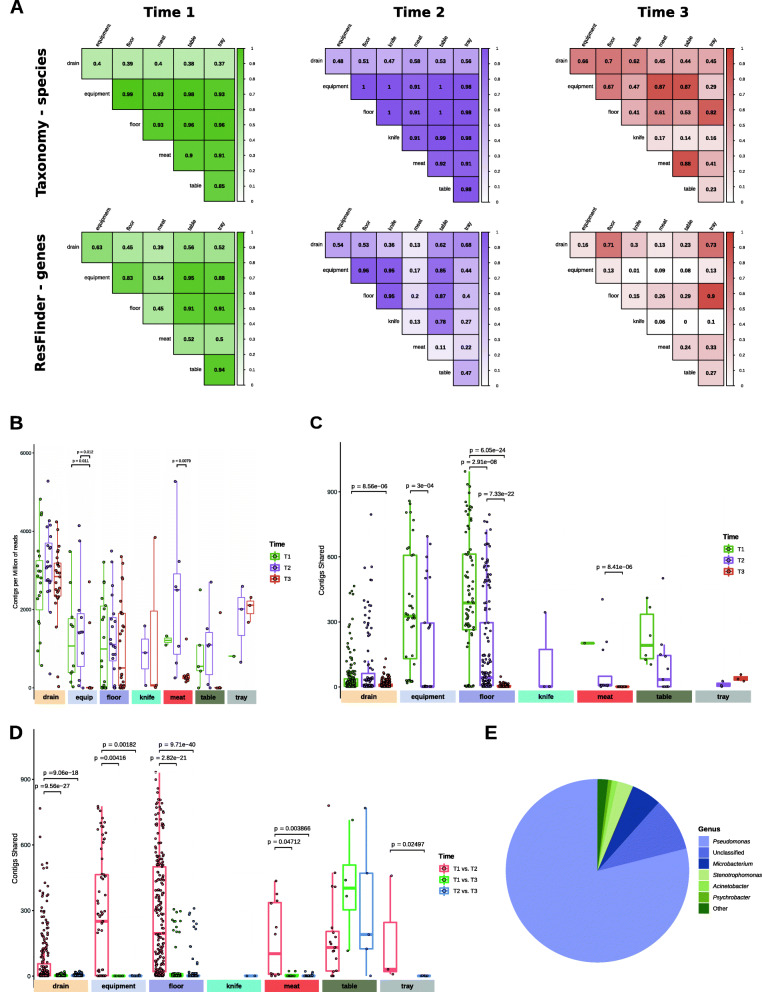
Microbial persistence in FPE. **A** Correlograms performed using average values, per surface type and sampling time, of each species and ARGs found by using kraken2 and ResFinder, respectively. Pearson correlation values are indicated within the cells. **B** Number of contigs with length > 1500 bp obtained on each FPE sample (*n* = 210). **C** Average values of contigs shared between samples from the same sampling time by surface type. **D** Average values of contigs shared between samples from 2 different sampling times by surface type. Only significant *p* values (*p* < 0.05) obtained from the Wilcoxon signed-rank test analysis are indicated. **E** Taxonomical classification of shared contigs. The proportions in the circle indicate the percentage of contigs assigned to each genus

We calculated the resemblance, at taxonomic species and ARG levels, of samples taken from different surfaces during the same sampling period (T1, T2, or T3) by calculating the Pearson correlation coefficient of the average values per surface and time. The resemblance of samples from different surface types decreased along time, at both taxonomic and ARG levels. Drains for taxonomy and drains and meat for the resistome had the lower correlation values at T1 and T2 (Fig. 7A). Likewise, knife samples for taxonomy and knives and equipment for the resistome showed the lowest correlation values at T3 (Fig. 7A). We also assessed the number of contigs shared between each pair of samples by using a very strict cut-off (blastn; 100% identity; coverage > 80%; contig length > 1500 bp). Although the total number of contigs was higher in T3 than in T1 and T2 (Fig. 7B), the number of contigs shared between samples was significantly lower (*p* < 0.001) at T3, as compared with those at T1 and T2, and this was observed for almost every surface (Fig. 7C). Similarly, although drains contained the highest number of total contigs (Fig. 7B), they had fewer shared contigs than other surfaces (Fig. 7C). When samples from different sampling times were compared, a significantly higher amount of contigs were shared between T1 and T2 samples than between those from T3 and other times, except for tables (Fig. 7D). Up to 79.0% of the shared contigs were assigned to *Pseudomonas*, 5.3% to *Microbacterium*, 2.8% to *Stenotrophomonas*, 1.0% to *Acinetobacter*, and 0.7% to *Psychrobacter.* These were the only genera linked to at least a 0.5% of the shared contigs (Fig. 7E).

## Discussion

The microbiome mapping activities revealed that FPE were dominated by a limited group of environmental bacteria, such as *Pseudomonas*, *Acinetobacter*, *Psychrobacter*, *Stenotrophomonas*, *Brevundimonas*, *Acidovorax*, or *Microbacterium*, among others, which constituted a core microbiota that was generally shared among different surfaces and rooms but evolved temporally.

Studies based on culture-independent methodologies have previously shown that hundreds of different species can be present in a single processing facility, but only a few taxa of residential bacteria commonly dominate FPE, as reviewed by Møretrø and Langsrud [1]. While the vast majority of the principal microbial taxa detected in our study have been frequently reported as dominant taxa in other previous studies characterizing food processing plants [1], our study is novel by virtue of providing a deep insight into how the microbiome evolves in a newly built FPE. Throughout the 1.5-year study, a more heterogeneous taxonomic profile across surface types and processing rooms was observed over time. *Pseudomonas*, which was the most abundant genus overall, became less dominant over time, although it was still the main group on drains, meat, equipment, and tables at T3. Notably, *Pseudomonas* is a taxonomic group of great interest in meat industries due to the recognized role of some *Pseudomonas* species as major meat spoilers [27] and is among the most frequently reported taxa found after sanitation of processing surfaces from all types of food production chains, with its persistence being likely due to low growth requirements and the ability to grow at low temperatures, form biofilms, and tolerate biocides [1]. Nevertheless, throughout the study, other taxa of the core microbiota were also prominent (e.g., at T3, *Psychrobacter* and *Acinetobacter* were the most abundant taxa on floors and trays, and on knifes, respectively).

Total abundance, alpha diversity, and β-dispersion of ARGs; abundance of ARGs conferring resistance to aminoglycosides, tetracyclines, and antibiotics of the MLSP group; and relative abundance of contigs associated with plasmids, were all significantly higher at T2, and especially at T3, than at T1. This, together with the higher occurrence of microorganisms showing resistance to certain CIA at T2, just after pork cutting activities started, suggests that pig carcasses and pork meat are a source of AR bacteria, ARGs, and MGE. Indeed, those ARG classes prevailing in T2 and T3 mainly confer resistance to the antibiotics most frequently used on pig farms [28] and a recent resistome study carried out at pig farms from the same region identified ARGs from the tetracyclines, aminoglycosides, and MLSP classes as the most prevalent in fecal, environmental, and slurry samples [29]. Moreover, some FCS, such as equipment, showed towards the end of the study a higher ARG CPM and a greater abundance of ARGs linked to resistance against CIA, and the rooms where more intense meat manipulation takes place (e.g., cutting rooms and rooms for post-cutting activities—R3 to R7) also had, in general, higher ARG CPM. This again suggests the likely transfer of resistant bacteria from carcasses and meat to FPE.

Although pig carcasses seem to be the main means of entry of AR bacteria and ARGs, daily activities at the processing plant may also shape the resistome. In this regard, drains exhibited the highest microbial loads (see Additional file 8), diversity and richness of taxa and ARGs, and abundance of integrons and LGT events. Drains represent complex microbial ecosystems where several factors that may favor the emergence and spread of AMR converge (i.e., they are environments with high humidity and contain dense microbial populations exposed to high contents of organic matter and run-off from meat processing activities, including low concentrations of cleaning and disinfection agents) [30]. This microenvironment provides ideal conditions for biofilm formation and horizontal gene transfer. In practical terms, poor drain maintenance may lead to the pooling of water and, eventually, the recontamination of other FPE and ultimately the food with AR bacteria.

ARGs were mainly associated with specific members of the principal microbiota (e.g., *Acinetobacter*, *Pseudomonas, Aeromonas, Mycoplasma, Brevundimonas, Psychrobacter, Stenotrophomonas*), but also with some less abundant taxa (e.g., *Thermus*, *Morganella*, *Streptococcus*), which can therefore be also highly relevant in terms of AMR transmission in FPE. *Acinetobacter* stood out as the most frequent carrier of ARGs. This is particularly relevant as *Acinetobacter* is responsible for hospital-acquired infections caused by multidrug-resistant isolates [31], and some reports have previously concluded that raw meat represents a reservoir of multidrug-resistant *Acinetobacter* strains and may serve as a vector for their spread into community and hospital settings [32].

Whereas the total abundance of ARGs associated with resistance to CIA decreased over time (except for some FCS, such as equipment and tables), culture-dependent analyses showed a sharp increase in occurrence of ESBL-producing *Enterobacteriaceae* and VRE once the facility started to operate at full capacity. This demonstrates that the pork production chain can serve as a transmission route for resistant microorganisms of clinical relevance, as also shown in previous studies [33]. Interestingly, the determinants identified in culture-dependent analyses were not detected through whole metagenome sequencing, which demonstrates the importance of combining both approaches when studying FPE resistomes with a focus on clinically relevant genes and species. Furthermore, the species obtained through culture-dependent approaches, with the exception of *Pseudomonas*, were found in low abundance in the metagenomes (see Additional file 1: Figure S8). Therefore, a species-focused isolation, involving an enrichment step, significantly improved the resistome screening performed following the culture-independent metagenomics approach.

The frequent co-occurrence of some ARGs associated with resistance to different antimicrobial classes, or the isolation of *Enterococcaceae* with multidrug-resistance profiles, suggest that co-selection for resistance to various different antibiotics can occur on FPE of meat processing plants. MGE, which in our study were found in a higher percentage of contigs on drain samples and at T3, can play a relevant role in such co-resistance events [34].

The resemblance of samples from different surface types and processing rooms at taxonomic and ARG level was higher at the first sampling times, where a relatively high amount of contigs (mainly from *Pseudomonas*) were shared among samples, than later throughout the study. This suggests that while specific taxonomic groups colonized and became dominant in the facility, the lineages or strains that thrived in different ecological niches within the processing plant were very different from each other, also in terms of genetic repertoire. This reflects their likely higher adaptability to the particular microenvironments prevailing in the different FPE within the facility.

## Conclusions

To our knowledge, this is the first extended longitudinal study dedicated to characterizing the microbiome of the built environment of a newly opened food processing plant. In doing so, we have found that a number of environmental dwelling taxa colonized FPE and became dominant and that a certain degree of microbiome specialization took place depending mainly on the surface or environment being assessed. We have also demonstrated that an important disruption of the microbiome happened once meat-cutting activities started, with the introduction in the facility of carcass-associated AR microorganisms and ARGs conferring resistance to those antibiotics more frequently used on pig farms in the region. Finally, we have identified several environmental reservoirs of AMR within the pork cutting facility, among which drains and some FCS, such as equipment and table surfaces, were of special relevance.

## Methods

### Sampling strategy

The survey was conducted in a Spanish newly opened pork processing facility producing packed fresh pork meat. Environmental samples were collected on ten sampling dates between July 2017 and February 2019. The first sampling visit was carried out just after the building and equipment installation works finished and before any pork processing activity or sanitation procedure took place. Sanitation activities started before the second sampling visit, conducted a week later, while a small number of pig carcasses were for the first time processed on the day that the third sampling visit took place. In subsequent samplings, initially carried out weekly until the fourth visit, then monthly until the sixth visit, and finally quarterly, the facility was fully operational. In an attempt to better characterize the resident (non-transient) microbiome, all sampling visits took place before the start of the working day, when the FPE were still clean after routine cleaning and disinfection on the previous evening. The following rooms were sampled at each sampling visit: a chilling room for carcasses (R1), two cold storage rooms for carcasses (R2), a cutting room exclusively used for white pigs processing (R3), a cutting room exclusively used for Iberian pigs processing (R4), several cold storage rooms used for different types of meat cuts obtained at the cutting rooms (R5), a small trotters’ washing room (R6), and a packaging room (R7) (Fig. 1). Environmental swab samples were taken from different food contact surfaces (FCS), which included tables, trays, knives, equipment, or conveyor belts, and non-food contact surfaces (NFCS), which included drains, walls, floors, sinks, trolley wheels, and others, as well as from the surface of carcasses and meat cuts (Fig. 1; see Additional file 8).

### Sample collection

Samples were collected by using HydraSponge sterile sponge swabs pre-moistened with 10 mL of neutralizing buffer (3M, USA). When enough surface was available (e.g., floors, walls), a surface of ~ 1 m^2^ was sampled, by swabbing surfaces first horizontally, then vertically and finally diagonally, turning the swab around in between. For other surfaces, where swabbing 1 m^2^ was not possible (e.g., drains or knives), individual units (e.g., 1 drain or 1 knife) were swabbed. When swabbing, the bag opening was kept to the side to decrease airborne contamination. Once the swab was taken, the air in the bag was manually and carefully removed, and the bag was sealed. Bags for culture-dependent analyses contained one single swab for each room and surface sampled, while 5 different swabs for each room and sample category were pooled together in sampling bags used for culture-independent analyses. In total, 1374 swab samples (229 for culture-dependent analyses and 1145 for culture-independent analyses, the latter ones pooled in 229 composite samples) were collected and categorized as drains (drain: 29.3% of the samples), equipment in contact with meat (equipment: 15.3%), floors, walls and other non-food contact surfaces (floor: 29.3%), knives (knife: 3.1%), carcasses and meat surfaces (meat: 8.7%), tables and conveyor belts (table: 8.3%), and trays (tray: 6.1%).

Appropriate single-use disposable protective clothing (e.g. gloves, footwear, hairnets) were used during the sampling visits and gloves were changed after each sample was taken to avoid cross-contamination of samples. Sampling bags were then placed in a cooling box containing ice packs and transported to the laboratory within 2 h for sample processing.

### Culture-dependent analyses

In culture-dependent analyses, strains from *Enterobacteriaceae*, *Staphylococcus* spp., *Pseudomonas* spp., and *Enterococcus* spp. were isolated as frequent carriers of acquired antibiotic resistance of relevance in swine farms and FPE. Then, recovered isolates were screened for ESBL-associated (*Enterobacteriaceae* and *Pseudomonas* spp.; *bla*_CTX-M_*, bla*_SHV_), carbapenemase-associated (*Pseudomonas* spp.; *bla*_OXA-50_*, bla*_IMP_*, bla*_VIM_*, bla*_KPC-1_*, bla*_NDM-1_), methicillin (*Staphylococcus* spp.; *mecA*), and vancomycin (*Enterococcus* spp.; *vanA, vanB*) resistance pheno- and genotypes.

#### Microbial isolation

A primary enrichment step was carried out by suspending sponge swab samples in 100 mL of Buffered Peptone Water (BPW, Merck, Germany). After incubation at 37 ± 1 °C for 18–24 h, this primary enrichment solution was used to inoculate different agar media for the recovery of the targeted microbial groups.

Eosin methylene blue (EMB, Merck, Germany) agar plates were inoculated with a sterile loop (10 μL) and incubated at 37 ± 1 °C for 18–24 h for the isolation of *Enterobacteriaceae*, whereas KingB agar plates supplemented with cetrimide, fucidin, and cephalotin (CFC) (Merck, Germany) were loop inoculated (10 μL) and incubated at 30 ± 1 °C for 44–48 h for the isolation of *Pseudomonas* spp*..* Presumptive *Enterobacteriaceae* (black colonies with metallic glitter) and *Pseudomonas* spp*.* (green fluorescent colonies under the UV light) colonies were freshly inoculated on Brain Heart Infusion agar plates (BHI, Merck, Germany) and cultivated at 37 ± 1 °C for 18–24 h for their incorporation to the culture collection.

The isolation of *Staphylococcus* spp. was performed by inoculating 100 μL of the primary enrichment on Baird Parker agar plates supplemented with egg yolk tellurite (BP, Merck, Germany), followed by 18–24 h of incubation at 37 ± 1 °C. Then, black or grey colonies with or without clear halo were freshly inoculated on BHI agar plates, which were incubated at 37 ± 1 °C for 18–24 h for their incorporation to the culture collection.

For the isolation of *Enterococcus* spp., the primary enrichment samples were streaked with a sterile loop (10 μL) on Slanetz and Bartley agar plates (VWR International, Belgium), which were incubated at 37 ± 1 °C for 18–24 h. After incubation, the colonies were transferred using a membrane filter onto pre-warmed (44 ± 1 °C) bile esculin azide agar plates (BEA, Oxoid Ltd., UK), which were further incubated at 44 ± 1 °C for 4 h for biochemical confirmation of enterococci (colonies producing a black precipitate). Then, presumptive *Enterococcus* spp. colonies were freshly inoculated on BHI agar plates, which were incubated at 37 ± 1 °C for 18–24 h for their incorporation to the culture collection.

The culture collection, which was stocked in cryoinstant tubes (VWR International, Belgium) at – 20 °C, was comprised of one single isolate per indicator microorganism and analyzed sample, which provided a culture collection of 360 isolates. All stocked isolates were analyzed through MALDI-TOF mass spectrometry (Microflex LRF, Bruker) for presumptive identity confirmation. For this, briefly, isolates were freshly inoculated on BHI agar plates, grown for 18–24 h at 37 ± 1 °C and one colony per strain was spread with a sterile toothpick on the surface of a well of the MSP96 Bruker steel plate. Then, 1 μL of matrix solution was added to each well and dried at room temperature in a laminar flow cabinet for 5 min. The Bruker BTS standard sample (mass calibration standard showing a typical *Escherichia coli* DH5 alpha peptide and protein profile plus additional proteins) was inoculated in the first well of the steel plate. The spectra interpretation was achieved by using the software MALDI Biotyper with the commercial spectra reference library provided by Bruker Daltonics.

#### Antibiotic susceptibility testing and identification of ARGs

*Enterobacteriaceae* and *Pseudomonas* spp. isolates from the culture collection were grown in BHI broth for 18–24 h at 37 ± 1 °C and then streaked on CHROMagar™ ESBL (CHROMagar, France) plates for the detection of ESBL-producing strains. *Pseudomonas* spp. isolates were also streaked on mSuperCarba™ (CHROMagar, France) plates for the detection of carbapenemase-producing strains. Chromogenic agar plates were in both cases incubated at 37 ± 1 °C for 18–24 h. Detection of ARGs commonly associated with ESBL (*bla*_CTX-M_ and *bla*_SHV_) and carbapenemase (*bla*_OXA-50_*, bla*_IMP_*, bla*_VIM_*, bla*_KPC-1_*,* and *bla*_NDM-1_) production phenotypes was achieved on grown isolates by PCR as previously described, with slight modifications (see Additional file 2: Table S4) [35-39].

*Staphylococcus* spp. isolates from the culture collection were grown in BHI broth for 18–24 h at 37 ± 1 °C and then streaked on MRSA Chromogenic Agar Base plates (Condalab, Spain), which were incubated at 37 ± 1 °C for 18–24 h, to detect methicillin-resistant strains. Detection of the *mecA* gene was achieved on grown isolates by PCR following the methodology previously described (see Additional file 2: Table S4) [40].

*Enterococcus* spp. isolates from the culture collection were grown in BHI broth for 18–24 h at 37 ± 1 °C and then inoculated on CHROMagar™ VRE plates (CHROMagar, France), which were incubated at 37 ± 1 °C for 18–24 h, to detect vancomycin-resistant strains. Detection of the *vanA* and *vanB* genes was achieved on grown isolates by conventional PCR following the methodology previously described (see Additional file 2: Table S4) [41, 42].

Amplification products were analyzed by electrophoresis in 1 × TBE buffer using 1.5% (w/v) agarose gels and detected by UV fluorescence after GelRed (Biotium Inc., CA, USA) staining, according to the manufacturer’s instructions. The PCR 100-bp Low Ladder (Sigma-Aldrich) was used as a molecular size marker.

In addition, a subset of 58 *Enterococcus* spp. isolates, representative of the different sampling times (T1, T2, and T3), were selected and their susceptibility to a wide range of antibiotics was determined through the microdilution method by using generic Sensititre *Enterococcus* EUVENC panels (Thermo Fisher Scientific, Oregon, USA) following the manufacturer’s instructions. After incubation of the panel plates at 37 ± 1 °C for 18–24 h, absence/presence of growth in each well was visually evaluated to calculate minimum inhibitory concentrations of each antibiotic. ECOFF values from EUCAST (or Clinical break points for Quinupristin.Dalfopristin, for which an ECOFF value was not available) were used as a threshold to rank *Enterococcus* isolates (*n* = 58) as resistant (non-wild type phenotype, according to EUCAST) or susceptible (wild type phenotype, according to EUCAST) to each antibiotic (see Additional file 2: Table S4) [43]. For *Enterococcus* isolates not belonging to *E. faecalis* or *E. faecium*, the thresholds to consider them as resistant or susceptible were fixed considering the highest ECOFF value from the two majority species of this genus (*E. faecalis* and *E. faecium*) in the EUCAST database. Isolates were considered multidrug resistant when they showed a non-wild–type phenotype against antibiotics from three or more different classes.

### Culture-independent analyses

#### DNA extraction

For the extraction of total metagenomic DNA, 10 mL of BPW was added to the composite swab samples, which were then homogenized for 1 min using a Stomacher lab blender. Subsequently, serial dilutions were spot plated (10 μL) on BHI agar plates, which were incubated at 30 ± 1 °C for 18–24 h, to enumerate the microbial load in each sample, while 10 mL aliquots were centrifuged at 6500 × g for 8 min to harvest the associated microbiota. The cell pellets were kept at – 20 °C until further use. The DNA was extracted from the pellets by using the DNeasy PowerSoil kit (Qiagen GmbH, Germany) following the manufacturer’s instructions, but conducting a double elution step with 25 μL of 10 mM Tris-HCl, in order to improve the DNA yield. DNA yields were measured with a Qubit fluorometer using the dsDNA HS assay kit (Invitrogen, Thermo Fisher Scientific, USA), while DNA quality was assessed by the 260/280 and 260/230 absorbance ratios determined by using a NanoDrop ND-1000 spectrophotometer (Thermo Fisher Scientific, Wilmintong, DE, USA). DNA quantity and quality values are shown on an additional file (see Additional file 8).

#### Library construction and shotgun sequencing

Extracted environmental DNA was employed to prepare the 150 bp paired-end sequencing libraries using the Illumina Nextera XTLibrary Preparation Kit (Illumina Inc., San Diego, CA, USA) using the method described by Rinke et al. [44]. Sequencing was performed on the Illumina NextSeq 500 platform using a NextSeq 500/550 High Output Reagent kit v2 (300 cycles), in accordance with the standard Illumina sequencing protocols.

#### Read quality filtering

Adapter removal and quality trimming of raw reads was performed using TrimGalore v 0.6.0 with default parameters (http://www.bioinformatics.babraham.ac.uk/projects/trim_galore/), a wrapper script for Cutadapt v2.6 [45] and FastQC v0.11.8 (http://www.bioinformatics.babraham.ac.uk/projects/fastqc/). The human and pig reference genomes, GRCh38 and Scrofa 11.1, respectively, were used to remove contaminant reads using Bowtie2 [46] v2.3.4.3 with default parameters. Resulting BAM files were processed using samtools [47] v1.9 and converted to FastQ format using bedtools [48] v2.27.1.

Samples with fewer than 200,000 reads were removed for further analyses. This cut-off value was selected since the negative controls always yielded a lower number of reads, and it allowed to retain a sufficient number of samples from all categories, thus allowing to extract solid conclusions. The discarded samples included the sequencing negative controls and 19 samples from the processing plant (2 from T1 and 17 from T3; 5 from equipment, 3 from floor, 4 from table, 4 from tray, and only 1 from drain, knife, and meat categories (see Additional file 1: Figure S8; see Additional file 8). Significant correlations were found between the number of reads obtained and both the microbial load and DNA amount (see Additional file 1: Figure S9).

#### Assembly into contigs and taxonomic annotation of reads and contigs

Each of the samples were independently subjected to *de novo* metagenomic assembly through metaSPAdes v3.13 [49] using default parameters. Filtered reads were taxonomically assigned by kraken2 v2.0.8-beta software [47] with the kraken2-microbial database (https://lomanlab.github.io/mockcommunity/mc_databases.html). Contigs longer than 1000 bp were taxonomically assigned using different approaches: (i) Kraken2 v2.0.8-beta software [50] with the kraken2-microbial database (https://lomanlab.github.io/mockcommunity/mc_databases.html); (ii) mmseqs2 software [51] with a database created with bacterial amino acid sequences extracted from the kraken2-microbial database by prodigal software [52], using as input the amino acid sequences for the coding regions in the contigs, extracted by prodigal; (iii) diamond [53] blastp with the same input and database employed for mmseqs2 analyses (see Supplementary File X for the command line pipeline employed). The *13.diamond_tax_filt.rb* and *14.mmseqs_tax_filt.rb* ruby scripts (https://github.com/JoseCoboDiaz/contig_taxonomy) were employed to filter the results obtained by mmseqs2 and diamond, which were screened to keep the genus assignment for a contig if at least the 90% of its coding regions were assigned to the same genus. Genus level classification of contigs obtained by at least 2 of the 3 approaches employed was kept for further analysis, by using the *15.bin_tax.rb* script (https://github.com/JoseCoboDiaz/contig_taxonomy). The same database was employed for the taxonomical assignment of both reads and contigs in order to avoid biases caused by the use of different databases. Only those reads belonging to the kingdom Bacteria were used for further analysis.

#### Antibiotic resistance gene annotation

The pipeline created for the annotation of ARGs is available at https://github.com/JoseCoboDiaz/ARG_bowtie_blast. Briefly, filtered reads were mapped against the ResFinder [54] database using Bowtie2 [46, 55], selecting the *--very-sensitive-local* parameter for bowtie2 alignment. The *.trimmed_pairs* fastq files generated by Bowtie2 were transformed into a fasta file where forward and reverse reads were concatenated. This new fasta file was used to perform a BLAST [56] against the ResFinder database using a 90% identity cut-off and taking 100 hits (max_target_seqs) in order to avoid problems associated with BLAST use in local [57]. Only the first hit per sequence was kept for further analyses. The combination of bowtie2 plus BLAST was previously checked with the entire dataset, using --sensitive-local, --very-sensitive-local, --sensitive, and --very-sensitive parameters (these 2 last options corresponding to an end-to-end approach). It was observed that end-to-end approaches had a lower amount of false positive hits (checked by BLAST step) than local approaches, which use partial read alignments as positive hits. Moreover, the use of local approaches increased the number of detected ARGs, and their combination with a BLAST as a double-check step reduced the amount of false positives, which accounted up to 20% of all ARGs found by bowtie2 for a 90% identity BLAST cut-off value (see Additional file 1: Figure S10).

The document *phenotypes.txt* was downloaded from the ResFinder repository (https://bitbucket.org/genomicepidemiology/resfinder_db/src/master/) and manually curated in order to modify the *class* variable, grouping those genes that confer resistance to macrolides, lincosamides, streptogramins, and pleuromutilins into the MLSP class, and those that confer resistance to oxazolidinones into the oxazolidinone class. This last group included *cfr* genes, which confer resistance to phenicols, lincosamides, oxazolidinones, pleuromutilins and streptogramins, the *optrA* gene, which confers resistance to phenicols and oxazolidinones, and the *poxtA* gene, which confers resistance to phenicols, oxazolidinones, and tetracyclines (see Additional file 9). This manually curated version of *phenotypes.txt* was used to create gene abundance and antibiotic resistance class abundance matrices from the blastn-firsthit file. Abundance matrices were transformed to count per million reads (CPM) matrices for further analyses using an R-script (https://github.com/JoseCoboDiaz/counts2CPM).

Additionally, ARGs associated with resistance to critically important antibiotics (see Additional file 9, sheet2), which were selected according to the World Health Organization guidelines [26], were selected to study the abundance and distribution of such genes within FPE.

#### Taxonomic assignment of ARGs

Each *.trimmed_pairs* fastq file generated by bowtie2 alignment versus the ResFinder [54] database was remapped against the contig file generated from the same sample dataset, using again Bowtie2 [46, 55] v2.3.4 and the option “--*very-sensitive-local”*, in order to match each ARG-read to the contig where it was assembled.

Taxonomic assignments of ARG-carrying contigs were matched to the output file from blastn *vs* Resfinder database at read level obtained previously. This new blast output file, with ARG and taxonomic information per read, was used to quantify ARGs and AMR classes per taxonomic group at genus level. Abundance matrices were transformed to CPM matrices for further analyses.

#### Mobilome analysis

Only contigs longer than 1000 bp were kept for the mobilome analysis. Using the assembled contig files as query files, plasmids were predicted by Plasflow [58], lateral gene transfer (LGT) events were detected by WAAFLE (https://huttenhower.sph.harvard.edu/waafle), and integrons were predicted by Integron_Finder [59].

Using their coordinates in the contigs, coding sequences (CDS) within LGT and integron regions were extracted from WAAFLE and Integron_Finder output files by using in-house ruby scripts and bedtools [48] utilities (https://github.com/JoseCoboDiaz/ARG-contig_mobilome_analysis). The extracted CDS fasta files were used for BLASTn comparison against the ResFinder database [54] using a 90% identity cut-off.

#### Analysis of shared contigs

All contigs longer than 1500 bp were combined in a single multi-fasta file, and an all-against-all search was performed with strict cut-offs of 100% identity and > 80% query coverage using BLASTn v2.8.1. Self-hits were removed, and the number of hits was averaged across pairwise combinations of samples.

#### Statistical analysis

The alpha diversity values of species and ARG richness and the Simpson diversity indices, for both taxonomy and resistome data, were calculated using the R package *vegan*. Comparisons of alpha diversity indices were carried out with the Wilcoxon signed-rank test through the R package *ggpubr* [60]. β-diversity was estimated by Principal Coordinates Analysis using Bray–Curtis dissimilarities and the *vegdist* function. Within-group dispersion was evaluated through the *betadisper* function. Both functions are located in the R package *vegan*. Finally, the effects of sampling time, surface type, and processing room on sample dissimilarities were determined by permutational multivariate analysis of variance using distance matrices (PERMANOVA) with the *adonis* function in the R package *vegan*. The *compare_means* function in the R package *ggpubr* was used to include statistically significant differences on boxplot figures, which were plotted by using the R package *ggplot2*.

Comparisons between multiple group samples for taxa, ARGs, resistome taxonomy, and mobilome data, including alpha diversity and total amount of ARGs, were performed by using the Kruskal–Wallis test and the *post hoc* Wilcoxon signed-rank test. *p* values were adjusted through the Benjamini & Hochberg [61] method, and significance was established at *p* < 0.05.

Correlation analyses were performed using the Pearson correlation coefficient, calculated with the *rcorr* function in the R package *Hmisc* [62]. Correlograms for the analysis of microbial persistence on FPE were performed using the average relative abundance values per surface and time group obtained for both species and ARGs.

Statistically significant differences among proportions of phenotypically resistant isolates depending on sampling time, processing room, and surface type were assessed through the two-proportion Z test using the *prop.test* function from *rstatix* R package applying Yates continuity correction. Statistically significant differences over time in the proportion of *Enterococcus*-resistant isolates to the different antibiotics tested were analyzed using the Fisher’s exact test with the function *pairwise_fisher_test* from *rstatix* R package.

All analyses were carried out using R version 3.6.2 [63].

## Supplementary Information

**Additional file 1: Figure S1.** Changes in diversity indices along sampling visits.. **Figure S2.** Changes in α-diversity indexes along time on surface and room sample groups. **Figure S3.** Differences in α- and β-diversity indices between different surfaces or rooms sampled at the same time point. **Figure S4.** Changes in relative abundance of the 6 main genera found on FPE samples along time within the same surface type. **Figure S5.** Diversity differences on resistome dynamics between different surfaces or sampled rooms from the same time point. **Figure S6.** Resistome antibiotic families and genes composition along sampled room and surface types. **Figure S7.** Characterization of the isolates culture collection. **Figure S8.** Relative abundance of reads belonging to species screened on culture-dependent approach. **Figure S9.** Number of reads obtained. **Figure S10.** Bowtie2 parameters comparison.

**Additional file 2: Table S1.** Adonis values. **Table S2.** Statistical analysis for ARGs associated with Critically Important Antibiotics (CIA). **Table S3.** Antibiotic concentration thresholds (μg/mL) for the categorization of Enterococcus isolates as resistant or sensitive. **Table S4.** Primers used for the detection by PCR analysis of isolates harboring different ARGs.

**Additional file 3. **Statistical analysis for taxonomy. The first column indicates the genus to be compared among samples. Next columns (until the column titled *Kruskal p-value*) indicate average values for each sample group being compared. Kruskal *p*-value columns indicate the *p*-values for the comparison between sample groups, which are presented for each surface type and processing room in the sheets *surface_time* and *room_time* respectively. The subsequent columns indicate *p*-values from the Wilcoxon pair-wise test for each pair of sample groups, which are also indicated on the column head. Statistical analyses performed by sampling time are presented on the *time* sheet, by sampling time for each surface type on the *surface_time* sheet, by surface type for each of the three time groups on the *TX_surface* sheets, by sampling time for each processing room on the *room_time* sheet, and by processing room for each of the three time groups on the *TX_room* sheets. NaN means *not a number*, and indicates those cases where statistical analyses could not be performed due to zero values on each sample to be compared.

**Additional file 4. **Statistical analysis for ARG classes. The first column indicates the ARG class to be compared among samples. Next columns (until the column titled *Kruskal p-value*) indicate average values for each sample group being compared. Kruskal *p*-value columns indicate the *p*-values for the comparison between sample groups, which are presented for each surface type and processing room on the sheets *surface_time* and *room_time* respectively. The subsequent columns indicate *p*-values from the Wilcoxon pair-wise test for each pair of sample groups, which are also indicated on the column head. Statistical analyses performed by sampling time are presented on the *time* sheet, by sampling time for each surface type on the *surface_time* sheet, by surface type for each of the three time groups on the *TX_surface* sheets, by sampling time for each room on the *room_time* sheet, and by processing room for each of the three time groups on the *TX_room* sheets. NaN means *not a number*, and indicates those cases where statistical analyses could not be performed due to zero values on each sample to be compared.

**Additional file 5. **Statistical analysis for ARG and taxonomical assignments. Columns from C to H indicate average values for ARG classes and taxonomic assignments per sampling time and food contact status (NFCS: non food contact surfaces; FCS: food contact surfaces). Columns I and J indicate *p*-values obtained from Kruskal-Wallis analyses within FCS and within NFCS samples, respectively, to analyze significant differences across time. Columns from K to P indicate *p*-values from Wilcoxon pair-wise tests performed for each pair of Time samples within NFCS and within FCS samples. Columns from Q to S indicate *p*-values from Wilcoxon pair-wise tests performed for NFCS versus FCS samples for each Time group. NaN means *not a number*, and indicates those cases where statistical analyses could not be performed due to zero values on each sample to be compared.

**Additional file 6. **Statistical analysis for ARGs. The first column indicates the ARGs to be compared among samples, while the second column indicates the ARG class they belongs to. Next columns (until the column titled *Kruskal p-value*, not included) indicate average values for each sample group being compared. Kruskal *p*-value columns indicate the *p*-values for the comparison between sample groups, which are presented for each surface type and each processing room on the sheets *surface_time* and *room_time* respectively. The subsequent columns indicate *p*-values from the Wilcoxon pair-wise test for each pair of sample groups, which are also indicated on the column head. Statistical analyses performed by sampling time are presented on the *time* sheet, by sampling time for each surface type on the *surface_time* sheet, by surface type for each of the three time groups on the *TX_surface* sheets, by sampling time for each processing room on the *room_time* sheet, and by processing room for each of the three time groups on the *TX_room* sheets. NaN means *not a number*, and indicates those cases where statistical analyses could not be performed due to zero values on each sample to be compared.

**Additional file 7.** LGTs-Integrons. Blastn results for those LGT and integron regions associated with ARGs. The first column indicates the contig name, while columns B to E indicate the origin of the sample where the contig was occurring (surface type, processing room, day of visit, sampling time). Column F indicates the best match obtained by blastn versus the ResFinder database, while columns G to P indicate the parameters of the alignment obtained by blastn. Columns Q to T show the hierarchical classification of the gene obtained as the best match hit, according to Supp. File 4. Column U indicates the genomic location obtained for the contig by the Plasflow pipeline. Columns V and W show the CladeA (host) and CladeB (source) for the lateral gene transfer (LGT) events detected. Rows highlighted in orange or yellow indicate contigs that contain more than one ARG within their integron region.

**Additional file 8. **Samples Metadata, DNA quantity and quality, reads and information on the culture-dependent approach. Columns from A to G indicate sample names together with their temporal and spatial location. Columns from H to N correspond to parameters related to the DNA extraction process and the total and filtered reads obtained after shotgun sequencing. Columns from O to Z detail the occurrence of different microorganisms in the culture dependent analyses, their phenotypic resistance to specific antibiotics, as revealed through their growth on selective agar plates, and the detection of ARGs. Resistant *Pseudomonas* spp. isolates harboring ARGs for β-lactams and carbapenems were not detected.

**Additional file 9. **Phenotypes table. Document *phenotypes.txt* downloaded from the ResFinder repository (https://bitbucket.org/genomicepidemiology/resfinder_db/src/master/) and manually curated in order to modify the *class* variable, grouping those genes that confer resistance to macrolides, lincosamides, streptogramins and pleuromutilins into the MLSP class, and those that confer resistance to oxazolidinones into the oxazolidinone class. This last group included *cfr* genes, which confer resistance to phenicols, lincosamides, oxazolidinones, pleuromutilins and streptogramins, the *optrA* gene, which confers resistance to phenicols and oxazolidinones, and the *poxtA* gene, which confers resistance to phenicols, oxazolidinones and tetracyclines.

## Data Availability

The data that support the findings of this study are available under NCBI Bioproject ID PRJNA656666, with BioSample accession numbers from SAMN15795042 to SAMN15795270.

## Abbreviations

FPE: Food processing environments
ARGs: Antibiotic resistance genes
AR: Antibiotic resistant
FCS: Food contact surfaces
NFCS: Non-food contact surfaces
BPW: Buffered Peptone Water
EMB: Eosin methylene blue
CFC: Cetrimide, fucidin, and cephalotin
BHI: Brain Heart Infusion
BP: Baird Parker
CPM: Counts per million reads
LGT: Lateral gene transfer
CDS: Coding sequences
MLSP: Macrolides, lincosamides, streptogramins, and pleuromutilins
CIA: Critically important antibiotics
MGE: Mobile genetic elements
ESBL: Extended-spectrum β-lactamase
VRE: Vancomycin-resistant *Enterococcaceae*
