# Supplementary material for: Microbial colonization and resistome dynamics in food processing environments of a newly opened pork cutting industry during 1.5 years of activity
Source: Microbiome. 2021 Oct 14;9:204. doi: 10.1186/s40168-021-01131-9 (PMC8515711; doi:10.1186/s40168-021-01131-9)
Supplement: Supplementary file 2 — Additional file 1: Figure S1. Changes in diversity indices along sampling visits.. Figure S2. Changes in α-diversity indexes along time on surface and room sample groups. Figure S3. Differences in α- and β-diversity indices between different surfaces or rooms sampled at the same time point. Figure S4. Changes in relative abundance of the 6 main genera found on FPE samples along time within the same surface type. Figure S5. Diversity differences on resistome dynamics between different surfaces or sampled rooms from the same time point. Figure S6. Resistome antibiotic families and genes composition along sampled room and surface types. Figure S7. Characterization of the isolates culture collection. Figure S8. Relative abundance of reads belonging to species screened on culture-dependent approach. Figure S9. Number of reads obtained. Figure S10. Bowtie2 parameters comparison. [file 40168_2021_1131_MOESM1_ESM.pdf]

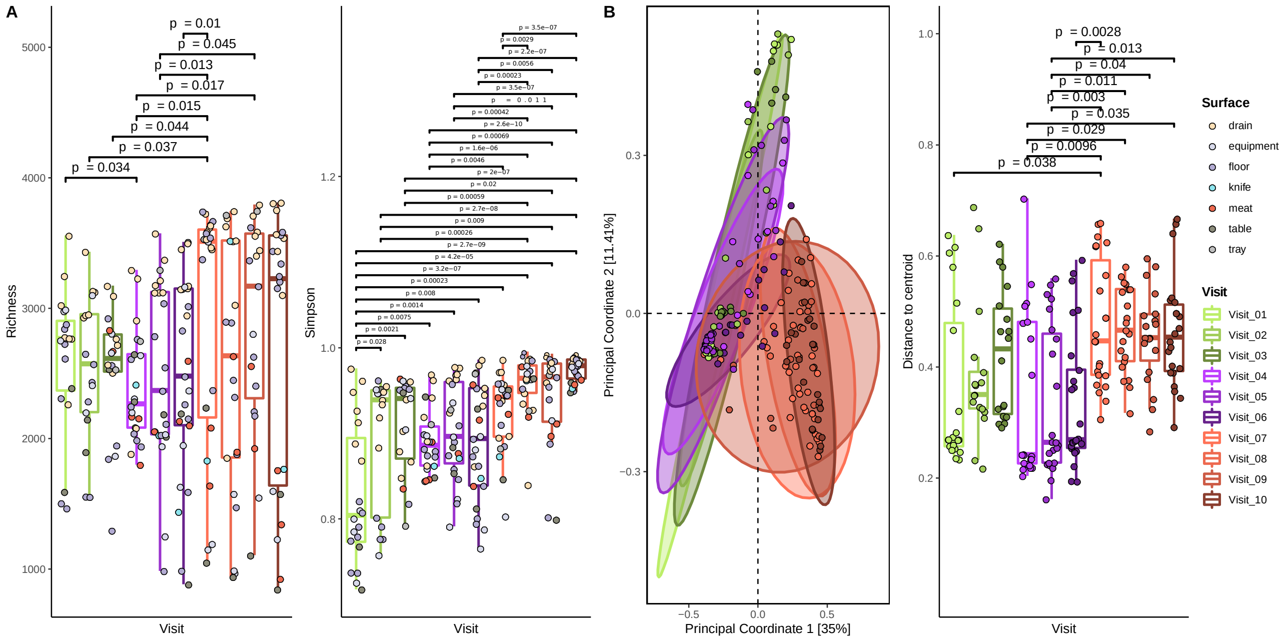

Figure S1. Changes in diversity indices along sampling visits.

A) Richness and Simpson indices at species level for the 210 FPE samples grouped by sampling visit. Indices were calculated from the relative abundance (%) matrix. B) Principal Coordinate Analysis, using Bray-Curtis distance, at species level with samples grouped by sampling visit. The centroid of each ellipse represents the group mean and the shape is defined by the covariance within each group. Distance to the centroid values were employed to evaluate the homogeneity of variances within each group. Only significant p-values (*p*<0.05) obtained from the Wilcoxon signed-rank test analysis are indicated. Visits are coloured according to the sampling time to which they belong (i.e., green for T1, purple for T2 and orange for T3).

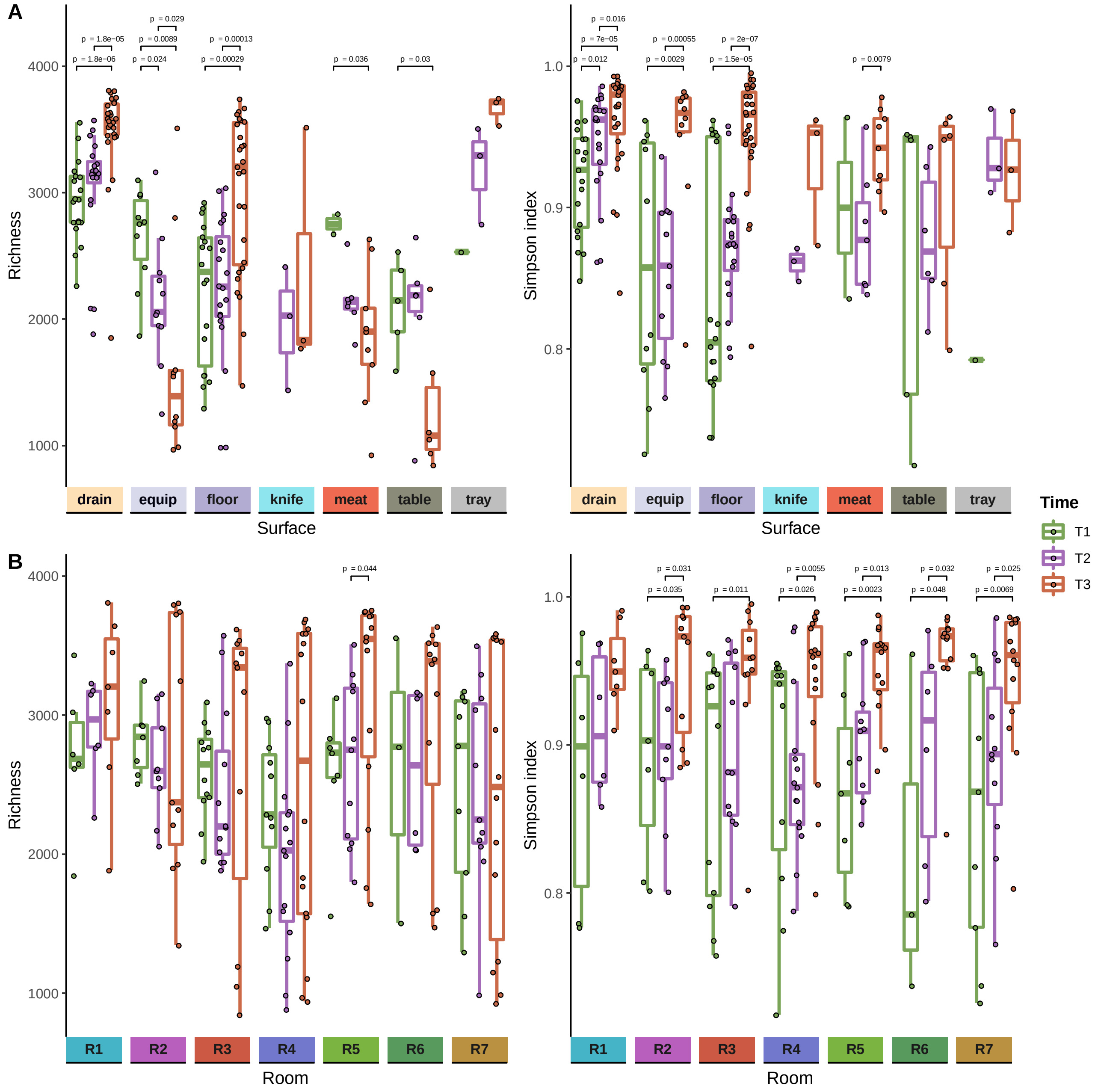

Figure S2. Changes in α-diversity indexes along time on surface and room sample groups..

Richness and Simpson indices at species level for the 210 FPE samples grouped by A) surface type and B) processing room. Indices were calculated from the relative abundance (%) matrix. Only significant p-values (p<0.05) obtained from the Wilcoxon signed-rank test analysis are indicated.

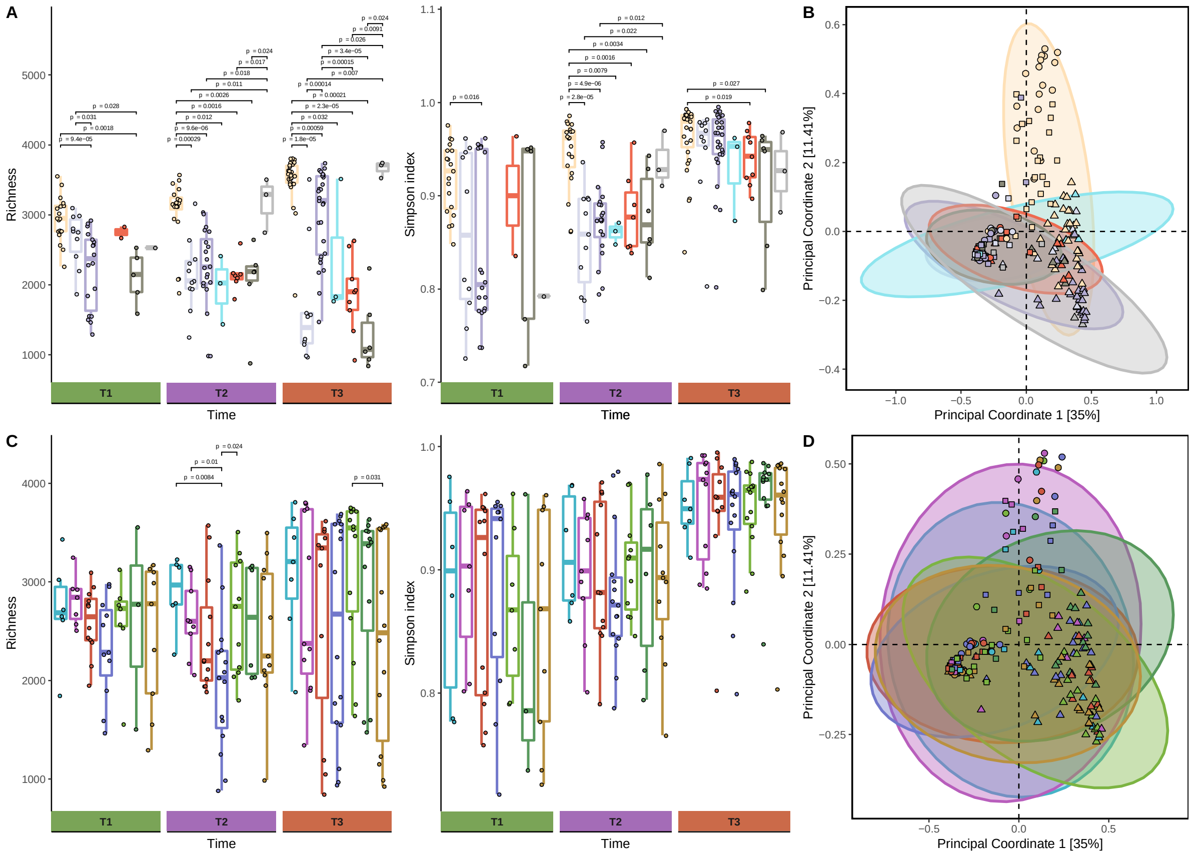

Figure S3. Differences in α- and β-diversity indices between different surfaces or rooms sampled at the same time point.

A) Richness and Simpson indices at species level for the 210 FPE samples grouped by A) surface type and C) sampled room within eac sampling time. Principal Coordinate Analysis, using Bray-Curtis distance, at species level with samples grouped by B) surface type and D) sampled room. The centroid of each ellipse represents the group mean and the shape is defined by the covariance within each group. Adonis test values are indicated in Table S1. Indices were calculated from the relative abundance (%) matrix. Only significant p-values (p<0.05) obtained from the Wilcoxon signed-rank test analysis are indicated.

Figure S4. Changes in relative abundance of the 6 main genera found on FPE samples along time within the same surface type.

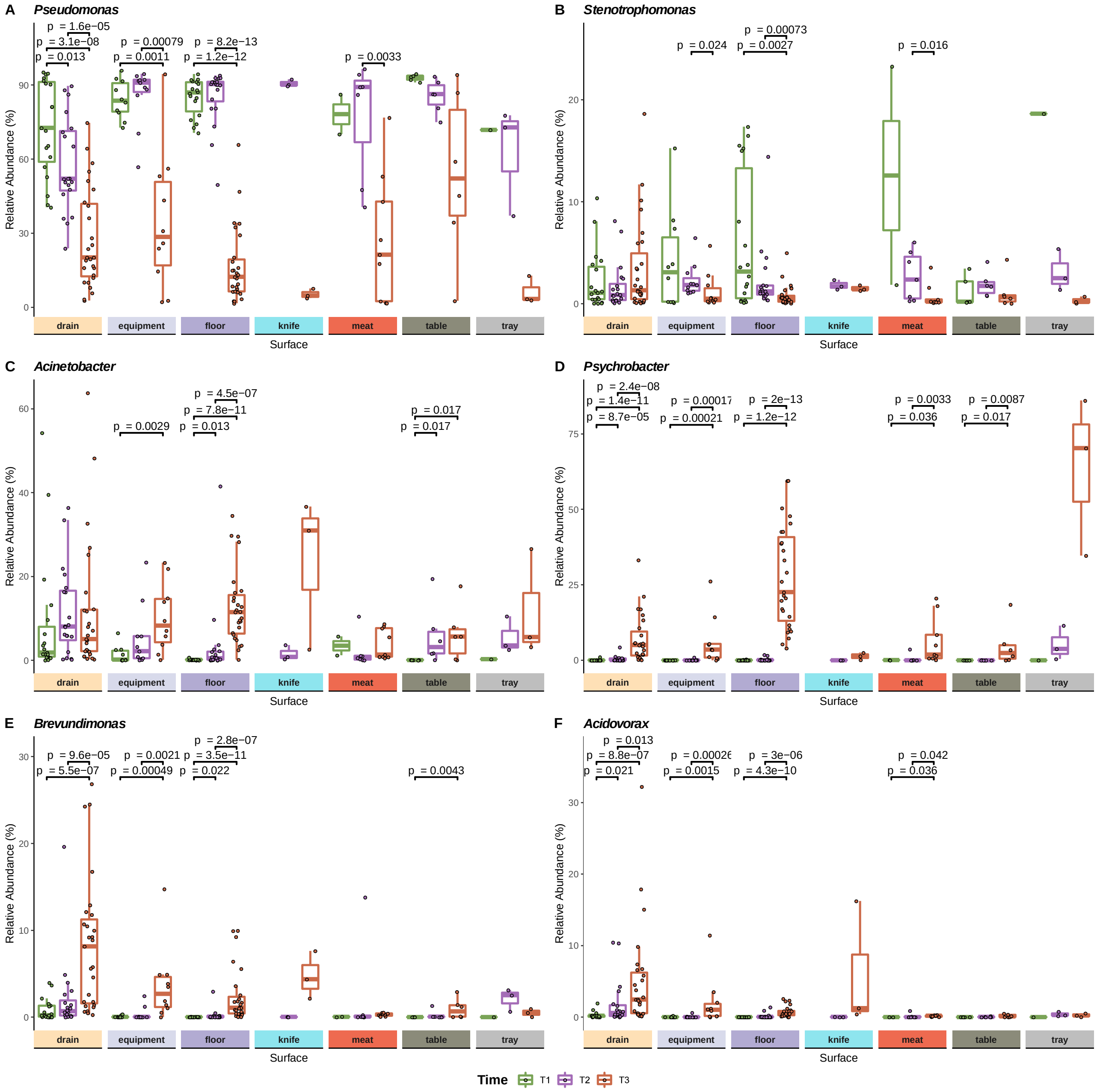

Boxplots of the 6 principal genera with significant changes in relative abundance over time, grouped by surface type. Genera represented are A) *Pseudomonas*, B) *Stenotrophomonas*, C) *Acinetobacter*, D) *Psychrobacter*, E) *Brevundimonas* and F) *Acidovorax*. Only significant p-values ($p<0.05$) obtained from the Wilcoxon signed-rank test analysis are indicated.

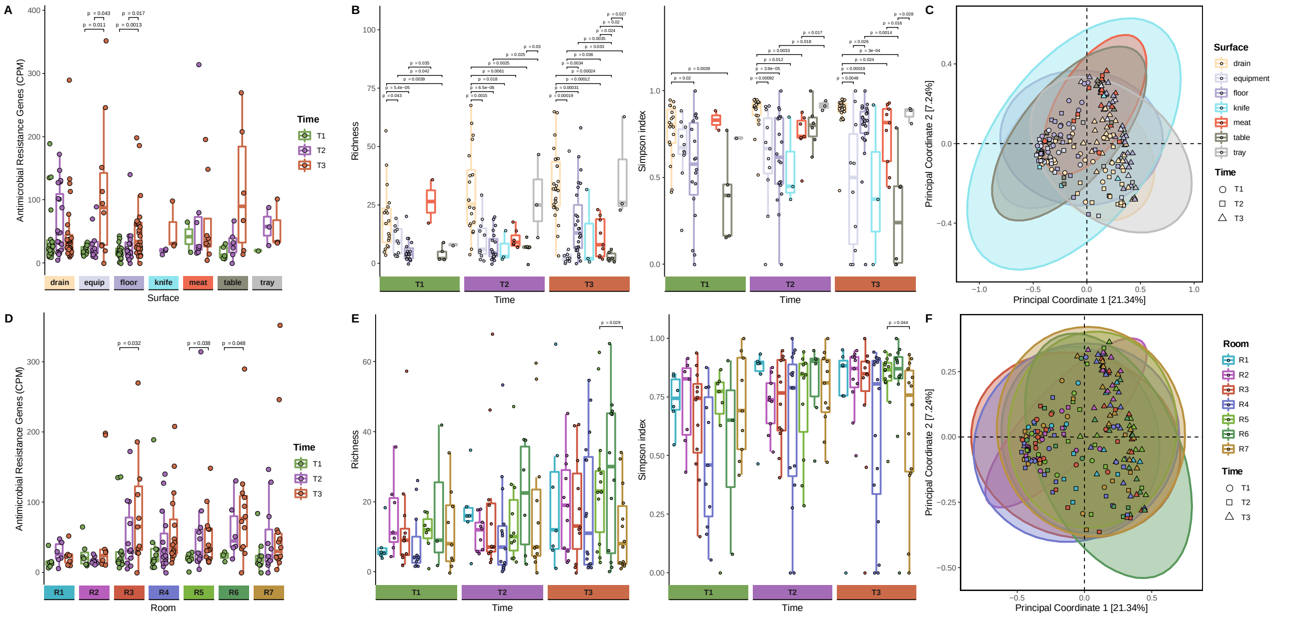

Figure S5. Diversity differences on resistome dynamics between different surfaces or sampled rooms from the same time point.

Antibiotic Resistance Genes (ARGs) load, in counts per million (CPM), detected on 210 FPE samples (n=55 for T1, n=70 for T2, n=85 for T3), represented by time among A) surface type and B) sampled rooms. Richness and Simpson indices calculated with the ARGs detected, represented by B) surface type and E) sampled room within each sampling time. Indices were calculated from the counts per million reads (CPM) matrix. Only significant p-values (p<0.05) obtained from the Wilcoxon signed-rank test analysis are indicated.

Principal Coordinate Analysis, using Bray-Curtis distance, at ARG level, with sample points grouped by C) surface type and F) sampled room. The centroid of each ellipse represents the group mean and the shape is defined by the covariance within each group. Adonis test values are indicated in Table S1.

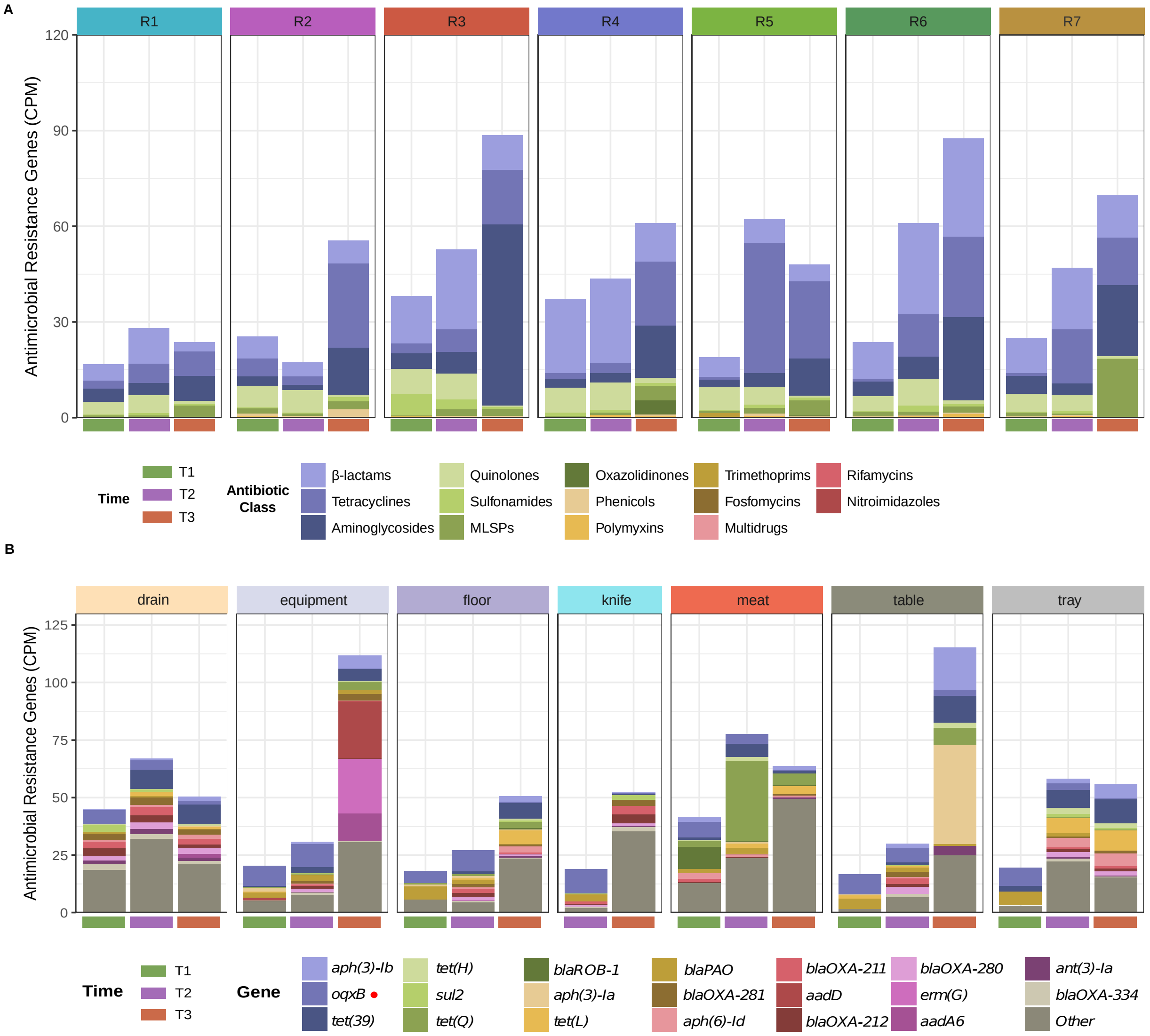

Figure S6. Resistome antibiotic families and genes composition along sampled room and surface types.

A) Barplot of the 14 ARG classes detected, calculated by summing ARG abundances according to the antibiotic classes they confer resistance to (Suppl. File 4). Each bar represents the average values for samples belonging to the same Room and Time groups, indicated at the top and the bottom of the plot, respectively. MLSP refers to macrolides-lincosamides-streptogramins-pleuromutilins.

B) Barplot of the twenty most abundant ARGs. Each bar represents the average values for samples belonging to the same Surface and Time groups, indicated at the top and the bottom of the plot,

respectively. MLSP refers to macrolides-lincosamides-streptogramins-pleuromutilins. ARGs associated with resistance to critically important antibiotics are marked with a red circle.

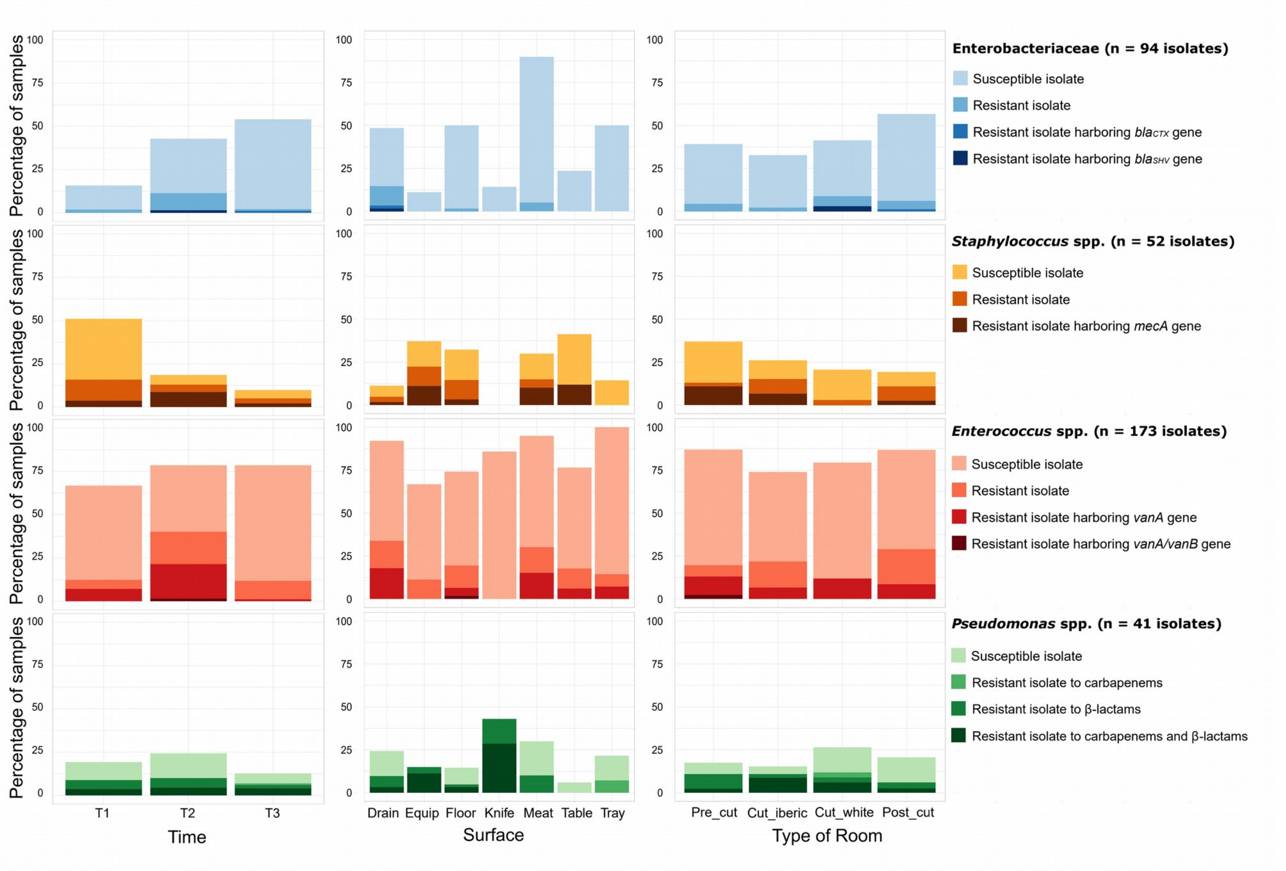

Figure S7. Characterization of the isolates culture collection

Proportion of samples containing antibiotic resistant (AR) isolates and ARGs from the culture-dependent analysis of the industry surfaces sampled (n=229). Colours from light to dark indicate samples where AR isolates were not detected, samples where AR isolates were detected but ARGs were not detected by PCR analysis, and samples where both AR isolates and ARGs were detected. Barplots were generated by grouping the samples by sampling time, surface type and processing room. Plots presented in rows are coloured by the microbial group (blue for *Enterobacteriaceae*, yellow for *Staphylococcus* spp., red for *Enterococcus* spp. and green for *Pseudomonas* spp.). Resistant *Pseudomonas* spp. isolates harboring ARGs for β-lactams and carbapenems were not detected.

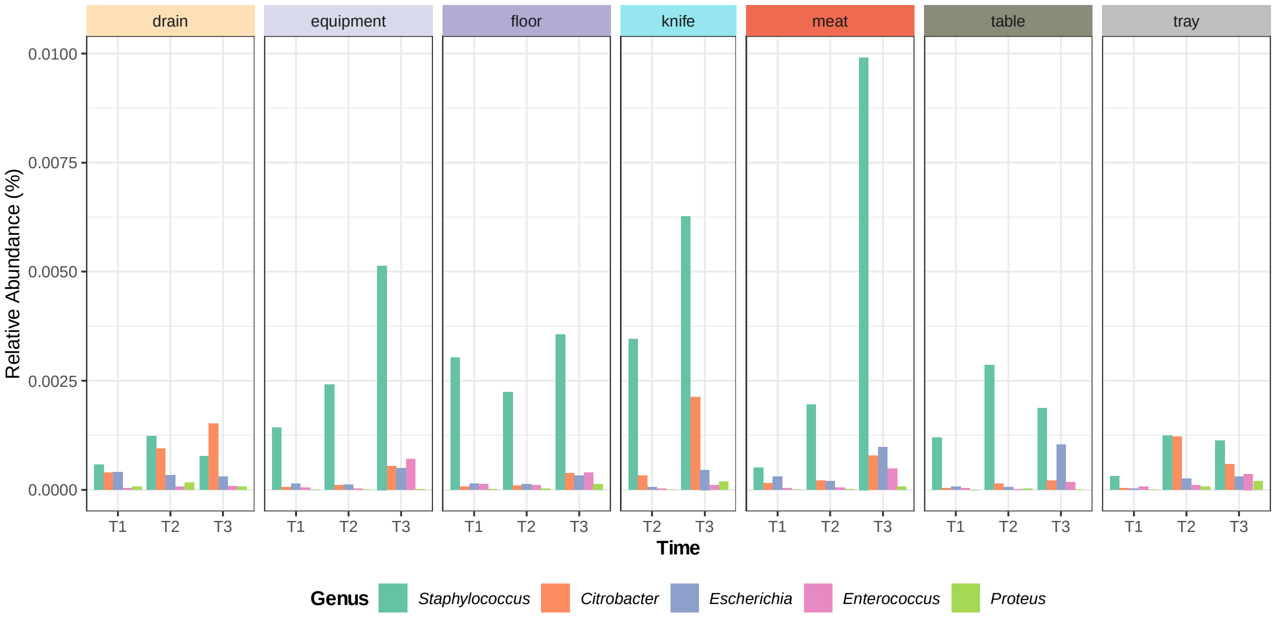

Figure S8. Relative abundance of reads belonging to species screened on culture-dependent approach.

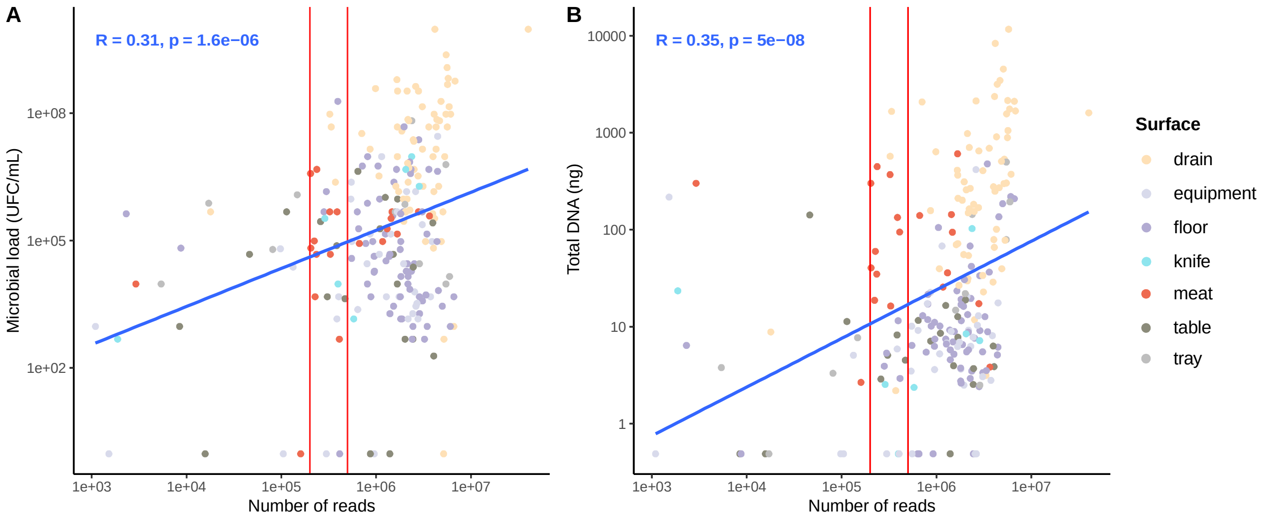

Figure S9. Number of reads obtained.

Number of reads obtained compared toA) microbial load measured (CFU/mL) and B) total DNA (ng) obtained for each of the 229 samples taken within the food processing plant. Microbial loads were obtained by spot plating on BHI agar plates. Total DNA was measured by using the Qubit fluorometer. Colours indicate the surface type from where the sample was taken. The red lines indicates the cut-off value of 200,000 reads, employed to discard samples with a low amount of reads, and 500,000 reads line, to indicate the high number of samples that could be lost if the cut-off values is increased.

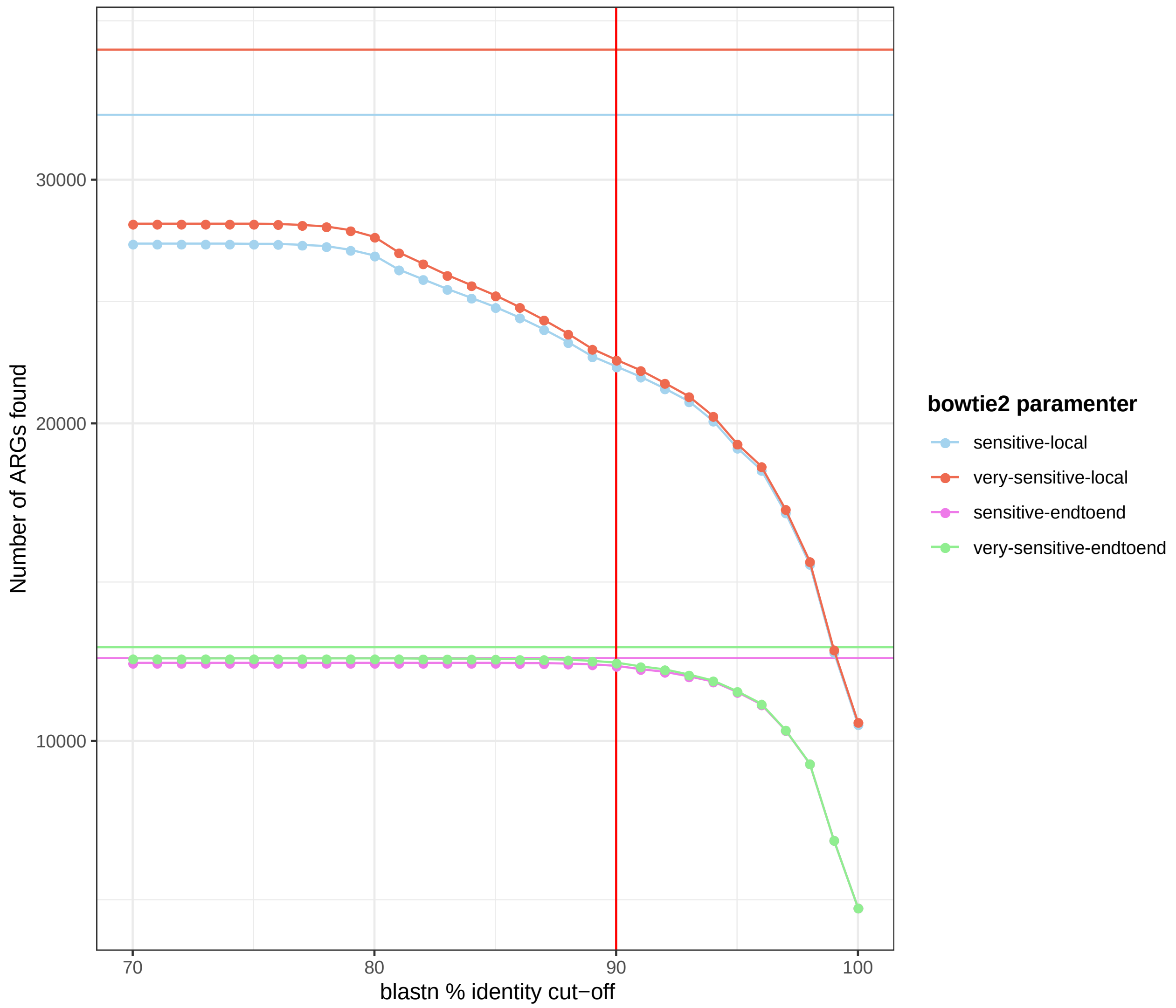

Figure S10. Bowtie2 parameters comparison.

Decrease of obtained BLAST hits versus ResFinder database by increasing the percent of identity cut-off, for the 4 different preset parameters available for bowtie2. Horizontal lines indicates the number of reads obtained by bowtie2 alignment without BLAST second step.
