# Supplementary material for: Microbial colonization and resistome dynamics in food processing environments of a newly opened pork cutting industry during 1.5 years of activity
Source: Microbiome. 2021 Oct 14;9:204. doi: 10.1186/s40168-021-01131-9 (PMC8515711; doi:10.1186/s40168-021-01131-9)
Supplement: Supplementary file 3 — Additional file 2: Table S1. Adonis values. Table S2. Statistical analysis for ARGs associated with Critically Important Antibiotics (CIA). Table S3. Antibiotic concentration thresholds (μg/mL) for the categorization of Enterococcus isolates as resistant or sensitive. Table S4. Primers used for the detection by PCR analysis of isolates harboring different ARGs. [file 40168_2021_1131_MOESM2_ESM.pdf]

Table S1. Adonis values. Results of Adonis analyses performed for taxonomy at species level, and for the resistome at ARG level. Up to 6 models were analyzed for each dataset, using the variables sampling time, surface type and processing room. NA means *not available*.

Taxonomy – Species level

	Df	Sums of Sqs	Mean Sqs	F.Model	R^2	Pr(>F)	
Time	2	11.93	5.97	30.59	0.228	0.001	***
Residuals	207	40.39	0.20	NA	0.772	NA	
Total	209	52.32	NA	NA	1.000	NA	
Surface	6	7.58	1.26	5.73	0.145	0.001	***
Residuals	203	44.74	0.22	NA	0.855	NA	
Total	209	52.32	NA	NA	1.000	NA	
Room	6	2.80	0.47	1.91	0.053	0.002	**
Residuals	203	49.52	0.24	NA	0.947	NA	
Total	209	52.32	NA	NA	1.000	NA	
Time	2	11.93	5.97	41.07	0.228	0.001	***
Surface	6	7.23	1.20	8.29	0.138	0.001	***
Time:Surface	11	5.55	0.50	3.47	0.106	0.001	***
Residuals	190	27.60	0.15	NA	0.528	NA	
Total	209	52.32	NA	NA	1.000	NA	
Time	2	11.93	5.97	31.60	0.228	0.001	***
Room	6	2.39	0.40	2.11	0.046	0.002	**
Time:Room	12	2.30	0.19	1.02	0.044	0.446	
Residuals	189	35.69	0.19	NA	0.682	NA	
Total	209	52.32	NA	NA	1.000	NA	
Surface	6	7.58	1.26	5.78	0.145	0.001	***
Room	6	2.46	0.41	1.88	0.047	0.006	**
Surface:Room	12	1.86	0.15	0.71	0.035	0.987	
Residuals	185	40.43	0.22	NA	0.773	NA	
Total	209	52.32	NA	NA	1.000	NA	

Resistome – Gene level

	Df	Sums of Sqs	Mean Sqs	F.Model	R^2	Pr(>F)	
Time	2	8.99	4.49	13.40	0.117	0.001	***
Residuals	202	67.75	0.34	NA	0.883	NA	
Total	204	76.74	NA	NA	1.000	NA	
Surface	6	6.17	1.03	2.89	0.080	0.001	***
Residuals	198	70.57	0.36	NA	0.920	NA	
Total	204	76.74	NA	NA	1.000	NA	
Room	6	3.83	0.64	1.73	0.050	0.002	**
Residuals	198	72.91	0.37	NA	0.950	NA	
Total	204	76.74	NA	NA	1.000	NA	
Time	2	8.99	4.49	14.67	0.117	0.001	***
Surface	6	5.83	0.97	3.17	0.076	0.001	***
Time:Surface	11	5.27	0.48	1.56	0.069	0.001	***
Residuals	185	56.66	0.31	NA	0.738	NA	
Total	204	76.74	NA	NA	1.000	NA	
Time	2	8.99	4.49	13.61	0.117	0.001	***
Room	6	3.47	0.58	1.75	0.045	0.002	**
Time:Room	12	3.54	0.29	0.89	0.046	0.856	
Residuals	184	60.75	0.33	NA	0.792	NA	
Total	204	76.74	NA	NA	1.000	NA	
Surface	6	6.17	1.03	2.95	0.080	0.001	***
Room	6	3.83	0.64	1.83	0.050	0.001	***
Surface:Room	12	3.92	0.33	0.93	0.051	0.713	
Residuals	180	62.83	0.35	NA	0.819	NA	
Total	204	76.74	NA	NA	1.000	NA	

Table S2. Statistical analysis for ARGs associated with Critically Important Antibiotics (CIA). Columns A, B and C indicate the average values of total amount of ARGs associated with CIA for each surface type and sampling time. Columns E, F and G indicate p-values from Wilcoxon pair-wise tests performed between samples from the same surface type and two different sampling times. The last column indicates the level of significance found in Wilcoxon pair-wise tests. NaN means *not a number*, and indicates those cases where statistical analyses could not be performed due to zero values on each sample to be compared.

Surface	T1	T2	T3	T1-T2 p.wilcox	T1-T3 p.wilcox	T2-T3 p.wilcox	
drain	8.01	11.70	3.01	0.550	0.000	0.000	****
equipment	9.89	10.72	31.27	0.468	0.049	0.034	*
floor	5.75	9.45	3.12	0.174	0.001	0.000	**
knife	-	10.77	0.56	-	-	0.077	
meat	9.83	8.34	8.25	0.500	0.509	0.907	
table	8.69	7.73	11.70	0.792	0.407	0.514	
tray	8.03	5.37	2.26	0.500	0.500	0.200	

Table S3. Antibiotic concentration thresholds (μg/mL) for the categorization of *Enterococcus* isolates as resistant or sensitive

The thresholds used were obtained from EUCAST ECOFF values, except in the case of quinupristin.dalfopristin, where the clinical break point was chosen due to the lack of an established ECOFF value. For *Enterococcus* isolates not belonging to *E. faecalis* or *E. faecium*, the threshold to consider them as resistant were fixed considering the highest ECOFF value from the two majority species of this genus (*E. faecalis* and *E. faecium*) in the EUCAST database.

	E. faecium	***E. faecalis***	***Enterococcus spp.***	***Measure***
Vancomycin	4	4	4	ECOFF
Teicoplanin	2	2	-	ECOFF
Quinupristin.Dalfopristin	4	-	-	Clinical breakpoint
Tetracycline	4	4	-	ECOFF
Daptomycin	8	4	-	ECOFF
Ciprofloxacin	8	4	-	ECOFF
Erythromycin	4	4	-	ECOFF
Tigecycline	0.25	0.25	-	ECOFF
Linezolid	4	4	-	ECOFF
Gentamicin	32	64	-	ECOFF
Ampicilin	4	4	-	ECOFF
Chloramphenicol	32	32	-	ECOFF

ECOFFs (μg/mL) retrieved from EUCAST: https://mic.eucast.org/Eucast2/

Clinical breakpoint for Quinupristin.Dalfopristin was retrieved from EUCAST:

https://www.eucast.org/fileadmin/src/media/PDFs/EUCAST_files/Breakpoint_tables/v_10.0_Breakpoint_Tables.pdf

Table S4. Primers used for the detection by PCR analysis of isolates harboring different ARGs.

Primer names, nucleotide sequences, amplicon sizes and annealing temperatures used to detect ARGs on the isolates belonging to the taxa indicated on column F. Asterisks indicate temperature modifications from the cited reference.

Primer name	Sequence(5'-3' direction)	Amplicon size	Annealing temperature	Isolates tested	Reference
bla-SHV.SE	ATG CGT TAT ATT CGC CTG TG	747 bp	56ºC	Enterobacteriaceae *Pseudomonas* spp.	[35]
bla-SHV.AS	TGC TTT GTT ATT CGG GCC AA				
CTX-M-U1	ATG TGC AGY ACC AGT AAR GTK ATG GC	593 bp	58ºC	Enterobacteriaceae *Pseudomonas* spp.	
CTX-M-U2	TGG GTR AAR TAR GTS ACC AGA AYC AGC GG				
bla_{KPC-1} -F	TGT CAC TGT ATC GCC GTC	879 bp	58ºC	*Pseudomonas* spp.	[36]
bla_{KPC-1} -R	CTC AGT GCT CTA CAG AAA ACC				
bla_{IMP} -F	GGA ATA GAG TGG CTT AAY TCT C	188 bp	52ºC	*Pseudomonas* spp.	[37]
bla_{IMP} -R	CCA AAC YAC TAS GTT ATC T				
bla_{VIM} -F	GAT GGT GTT TGG TCG CAT A	390 bp	52ºC	*Pseudomonas* spp.	
bla_{VIM} -R	CGA ATG CGC AGC ACC AG				
NDM-1gf	ACC GCC TGG ACC GAT GAC CA	264 bp	58ºC	*Pseudomonas* spp.	[38]
NDM-1gr	GCC AAA GTT GGG CGC GGT TG				
bla_{OXA-50} S	AAT CCG GCG CTC ATC CAT C	869 bp	60ºC	*Pseudomonas* spp.	[39]
bla_{OXA-50} AS	GGT CGG CGA CTG AGG CGG				
MecA1	GTA GAA ATG ACT GAA CGT CCG ATA A	310 bp	55ºC *	*Staphylococcus* spp.	[40]
MecA2	CCA ATT CCA CAT TGT TTC GGT CTA A				
AF	GCG CGG TCC ACT TGT AGA TA	314 bp	56.5ºC	*Enterococcus* spp.	[41]
AR	TGA GCA ACC CCC AAA CAG TA				
P1	CAT CGC CGT CCC CGA ATT TCA AA	297 bp	58ºC *	*Enterococcus* spp.	[42]
P2	GAT GCG GAA GAT ACC GTC GCT				
